# Ingenuity pathway analysis of *α*-synuclein predicts potential signaling pathways, network molecules, biological functions, and its role in neurological diseases

**DOI:** 10.3389/fnmol.2022.1029682

**Published:** 2022-11-29

**Authors:** Sharad Kumar Suthar, Sang-Yoon Lee

**Affiliations:** ^1^Neuroscience Research Institute, Gachon University, Incheon, South Korea; ^2^Department of Neuroscience, College of Medicine, Gachon University, Incheon, South Korea

**Keywords:** α-synuclein, ingenuity pathway analysis, canonical signaling pathways, biological functions and diseases, regulators, interactome, toxicity, neuroinflammation

## Abstract

Despite the knowledge that mutation, multiplication, and anomalous function of *α*-synuclein cause progressive transformation of *α*-synuclein monomers into toxic amyloid fibrils in neurodegenerative diseases, the understanding of canonical signaling, interaction network molecules, biological functions, and role of *α*-synuclein remains ambiguous. The evolution of artificial intelligence and Bioinformatics tools have enabled us to analyze a vast pool of data to draw meaningful conclusions about the events occurring in complex biological systems. We have taken the advantage of such a Bioinformatics tool, ingenuity pathway analysis (IPA) to decipher the signaling pathways, interactome, biological functions, and role of *α*-synuclein. IPA of the *α*-synuclein NCBI gene dataset revealed neuroinflammation, Huntington’s disease, TREM1, phagosome maturation, and sirtuin signaling as the key canonical signaling pathways. IPA further revealed Parkinson’s disease (PD), sumoylation, and SNARE signaling pathways specific to the toxicity of *α*-synuclein. A frequency distribution analysis of *α*-synuclein-associated genes from the NCBI dataset that appeared in the predicted canonical pathways revealed that NFKB1 was the most populated gene across the predicted pathways followed by FOS, PRKCD, TNF, GSK3B, CDC42, IL6, MTOR, PLCB1, and IL1B. Overlapping of the predicted top-five canonical signaling pathways and the *α*-synuclein NCBI gene dataset divulged that neuroinflammation signaling was the most overlapped pathway, while NFKB1, TNF, and CASP1 were the most shared molecules among the pathways. The major diseases associated with *α*-synuclein were predicted to be neurological diseases, organismal injury and abnormalities, skeletal and muscular disorders, psychological disorders, and hereditary disorders. The molecule activity predictor (MAP) analysis of the principal interaction network of *α*-synuclein gene SNCA revealed that SNCA directly interacts with APP, CLU, and NEDD4, whereas it indirectly communicates with CALCA and SOD1. Besides, IPA also predicted amyloid plaque forming APP, cytokines/inflammatory mediators IL1B, TNF, MIF, PTGS2, TP53, and CCL2, and kinases of MAPK family Mek, ERK, and P38 MAPK as the top upstream regulators of *α*-synuclein signaling cascades. Taken together, the first IPA analysis of *α*-synuclein predicted PD as the key toxicity pathway, neurodegeneration as the major pathological outcome, and inflammatory mediators as the critical interacting partners of *α*-synuclein.

## Introduction

**Figure 1:**
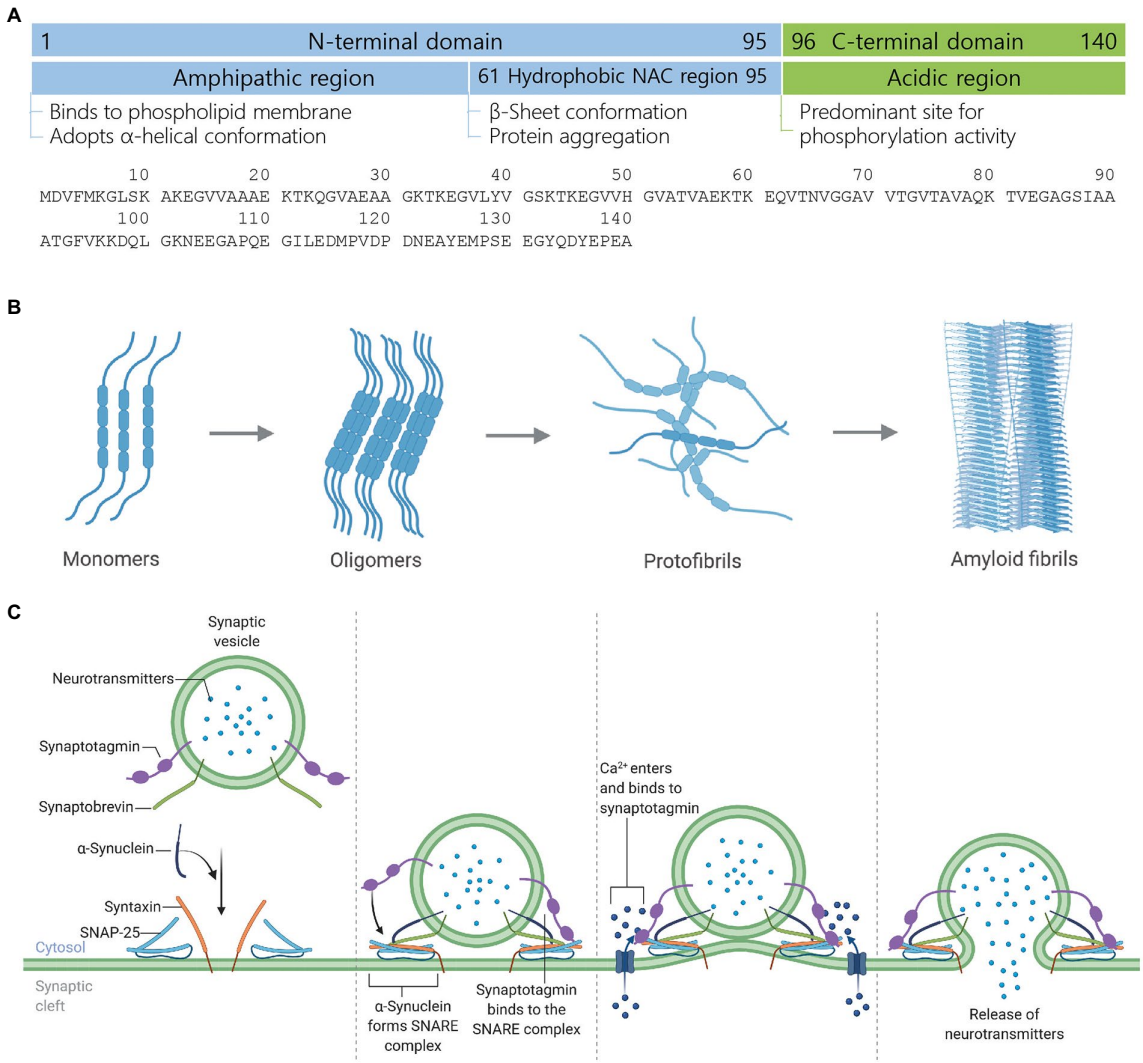
Structure, aggregate formation, and neurotransmission facilitation mechanism of *α*-synuclein. **(A)** Structure of *α*-synuclein highlighting different structural domains, their cardinal roles and an amino acid sequence. **(B)** Development of toxic *α*-synuclein aggregates. In the healthy brain, *α*-synuclein exists as a natively unfolded monomer. Upon mutation, multiplication, or unfavorable posttranslational modification, *α*-synuclein starts forming oligomers, which recruit further monomers and eventually form *β*-sheet-rich toxic amyloid fibrils. Amyloid fibrils form LB and LN, leading to neurodegeneration and cell death. **(C)** Mechanism of neurotransmission facilitation by *α*-synuclein. *α*-Synuclein is a presynaptic protein while synaptotagmin and synaptobrevin are synaptic vesicle membrane-associated proteins. During the docking of neurotransmitters-loaded synaptic vesicle onto the plasma membrane, *α*-synuclein binds to synaptobrevin while two other synaptic membrane proteins syntaxin and SNAP-25 are recruited to form the SNARE complex, which mediates vesicle priming. Action potential reaches the presynaptic terminal, calcium channel within the presynaptic plasma membrane opens, calcium ions enter into the cytosol, and bind to synaptotagmin, which leads to the exocytosis or release of neurotransmitters. Figure created with BioRender.com.

Alpha-synuclein (*α*-synuclein) is 140 amino acids (Nakajo et al., 1993; Uéda et al., 1993), intrinsically disordered monomer protein in normal cellular conditions (Weinreb et al., 1996). Structurally, *α*-synuclein protein has three distinct regions; (i) N-terminal domain (1–95 residues), which binds to phospholipid membrane and upon binding, adopts *α*-helical conformation (Uéda et al., 1993; Davidson et al., 1998; Bussell and Eliezer, 2003; Bussell et al., 2005; Vamvaca et al., 2009; Zarbiv et al., 2014), (ii) Non-amyloid component region (61–95 residues), which is also called central hydrophobic region is responsible for *α*-synuclein aggregation by adopting *β*-sheet conformation (Uéda et al., 1993; Conway et al., 2000; Serpell et al., 2000; Giasson et al., 2001; Tuttle et al., 2016), and (iii) C-terminal domain (96–140 residues) is rich of acidic residues and a major site for phosphorylation activity (Fujiwara et al., 2002; Anderson et al., 2006; Figure 1A).

During cellular stress and pathological conditions, *α*-synuclein monomers trigger the formation of dimers *via* increased interaction with each other, which upon continuous interactions transformed into oligomers (Lashuel et al., 2013). These oligomers further recruit monomers to form protofibrils and finally *β*-sheet-rich insoluble amyloid fibrils (Waxman and Giasson, 2009; Lashuel et al., 2013), which are major components of Lewy bodies (LB) and Lewy neurites (LN) in Parkinson’s disease (PD) and dementia with LB (DLB; Spillantini et al., 1997; Wakabayashi et al., 1997; Baba et al., 1998; Irizarry et al., 1998; Takeda et al., 1998; Trojanowski and Lee, 1998), and oligodendrocytes in multiple system atrophy (MSA; Arima et al., 1998; Spillantini et al., 1998; Tu et al., 1998; Wakabayashi et al., 1998; Figure 1B).

*α*-Synuclein acts as a chaperone for soluble NSF attachment protein receptor (SNARE)-complex assembly and upon recruitment to the SNARE-complex, facilitates the docking of synaptic vesicle onto the presynaptic plasma membrane. The fusion of synaptic vesicle to the presynaptic nerve terminals results in the release of neurotransmitters (Burré et al., 2010, 2014; Figure 1C). The deletion or knockout of *α*-synuclein in rodents promotes or exacerbates neurodegeneration, whereas the transgenic expression of *α*-synuclein rescued the animals from neurodegeneration (Chandra et al., 2005; Burré et al., 2010). Even though *α*-synuclein plays a critical role in neurodegeneration, its biological role in the initiation and progression of neurodegenerative diseases and disorders and the canonical signaling interactome remains elusive. Thus, the lack of information about *α*-synuclein’s biological functions, role, signaling, and interaction cohorts weakens our understanding of the molecular mechanism behind neurodegeneration as well as limits the development of therapeutics for early diagnosis and treatment of neurodegenerative diseases and disorders. In this work, we have made an attempt to reveal the canonical signaling interactome and biological functions of *α*-synuclein using the ingenuity pathway analysis (IPA)-based bioinformatics approach.

## Materials and methods

### Preparation of the *α*-synuclein NCBI gene dataset

*α*-Synuclein and associated genes were searched in the National Center for Biotechnology Information (NCBI) database with a search query “Alpha-synuclein.” The search was limited to the *Homo sapiens* genes only. A total of 215 *α*-synuclein-related genes available in the NCBI database till 26 August 2021 were downloaded as a text file (Supplementary Table S1; NCBI, 2021), which were subjected to analyzes on the IPA, QIAGEN.

### IPA expression analysis of the *α*-synuclein NCBI gene dataset

A text file of *α*-synuclein and associated genes (Supplementary Table S1) obtained from the NCBI database was imported onto the IPA server for a new “core analysis.” In the new core analysis, the “expression analysis” classification was selected. The default file format “flexible format” was chosen and the gene identification numbers (gene ID) were defined. For the population of genes to be considered for *P*-value calculations, the reference set was defined as the Ingenuity Knowledge Base genes only while for relationships that affect networks and upstream regulator analysis, both “direct and indirect relationships” were selected. For interaction networks, 35 molecules per network, 25 networks per analysis, and endogenous chemicals were selected while casual networks were predicted based on the score for master regulators for relationships to diseases, functions, genes, and chemicals. All node types, such as biological drugs, canonical pathways, all types of chemicals (endogenous, kinase, and protease inhibitors, drugs, toxic substances, and reagents), complexes, cytokines, diseases, enzymes, etc*.,* were marked for the core analysis. All data sources available on the IPA server were marked while the confidence of the prediction was limited to the experimentally observed values only. The species selection was restricted to humans only. For tissues and cells selection, tissues, cells, nervous system, organ systems, and other tissues and primary cells were marked. To observe the effect of possible mutations in IPA predictions, all types of mutations and effects, such as functional effect, inheritance mode, translation impact, unclassified mutations, zygosity, and wild type, were selected. The results were ranked based on Fisher’s exact test, where the smaller *P*-value indicates the higher significance of predicted results (IPA, 2021; Suthar et al., 2021). GraphPad Prism 6 was used to plot the graphs at various stages of the study.

### IPA toxicity analysis of *α*-synuclein, amyloid beta, tau, and Huntingtin

*α*-Synuclein, amyloid beta, tau, and Huntingtin *Homo sapiens* genes SNCA (gene ID 6622), APP (gene ID 351), MAPT (gene ID 4137), and HTT (gene ID 3064), respectively, were downloaded as an individual text file from the NCBI database (NCBI, 2022). IPA was performed by selecting the following options/parameters step-by-step available for selection on the IPA server (IPA, 2021); (i) the NCBI text file of the respective gene was imported onto the QIAGEN IPA server for a new core analysis, (ii) the imported text file was retained in the flexible format and the gene identification (GI) number of the SNCA/APP/MAPT/HTT was defined, (iii) IPA function “toxicity analysis” was chosen for the study, (iv) for the population of genes to be considered for *P*-value calculations, the reference set was limited to the Ingenuity Knowledge Base genes only, (v) for SNCA/APP/MAPT/HTT molecular network generation, we opted for molecules displaying both direct and indirect relationships in the network, (vi) furthermore, for SNCA/APP/MAPT/HTT molecular network generation, we opted for molecules exhibiting interaction networks as well as casual networks, (vii) an interaction network contained 35 molecules per network and 25 networks per analysis, (viii) the types of molecules to be included for network generation (referred to as node types), all node types, such as biological drugs, chemicals, complexes, cytokines, diseases, enzymes, etc. were selected, (ix) to consider the data sources for IPA predictions, all the data sources available on the IPA server were chosen, (x) to predict the relationships among the network molecules, the data sources with only experimentally observed molecular relationships were considered, (xi) IPA predictions were kept limited to the human species only, (xii) to opt for the data source experiments performed in the types of the organ system, tissues, and cell lines for IPA predictions, analysis was kept limited to the experimental results from tissues, cells, nervous system, and organ system only, (xiii) to consider the mutated molecules appearing in the network generation, all types of mutations, such as functional, inherited, translational, zygotic, and unclassified, were chosen, and (xiv) IPA prediction results were ranked based on the significance value (*P*-value) calculated by the right-tailed Fisher’s exact test (Suthar et al., 2021).

## Results

### IPA expression analysis of the *α*-synuclein NCBI gene dataset

#### Prediction of *α*-synuclein canonical pathways, interaction network molecules, and pathological implications

**Figure 2:**
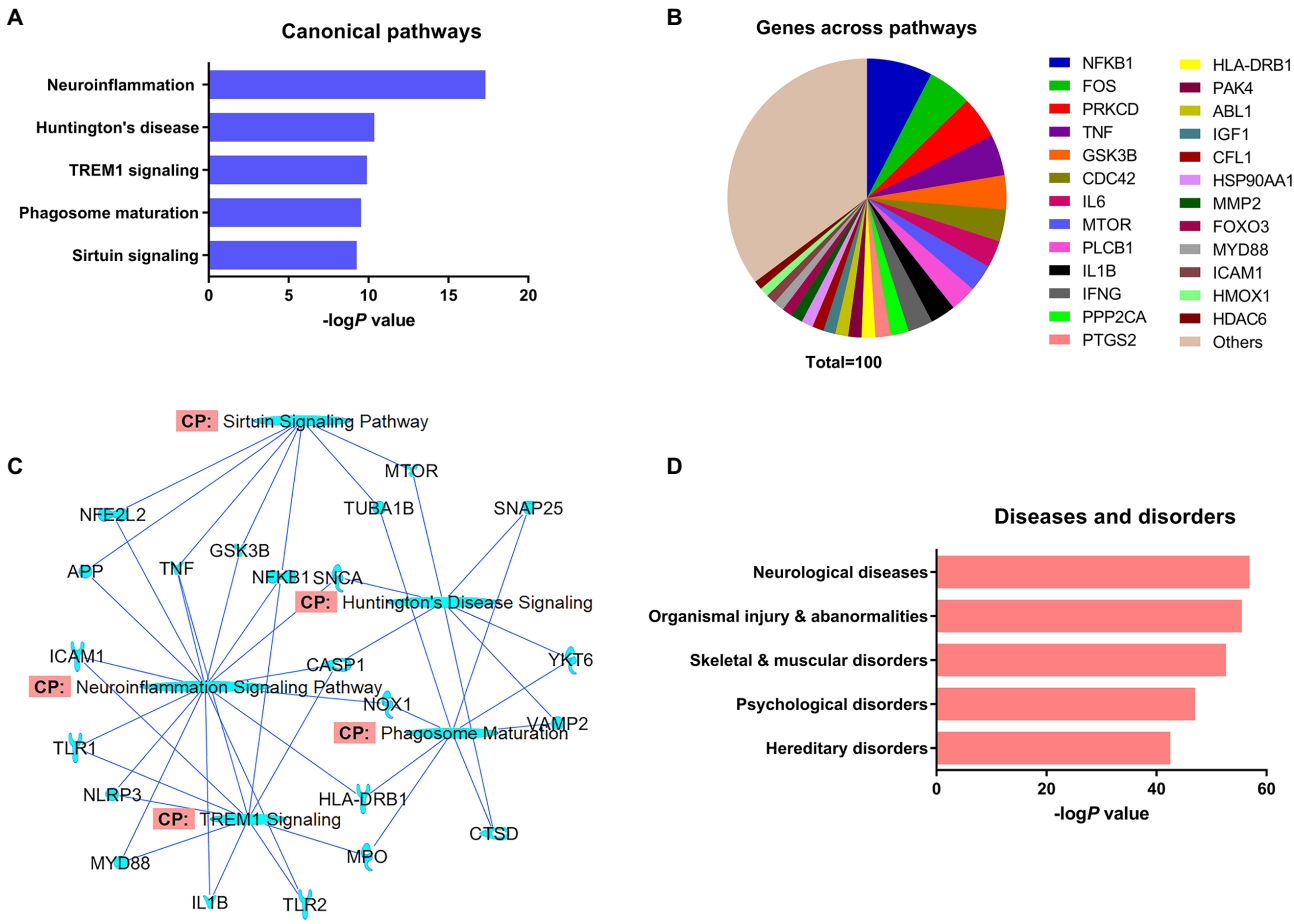
The expression analysis of differentially regulated *α*-synuclein and associated genes from the NCBI dataset by IPA. **(A)** Top-ranked canonical pathways of *α*-synuclein. **(B)** Significantly populated *α*-synuclein-associated genes from the NCBI dataset that appeared in the predicted canonical pathways. **(C)** Overlapping of the top-ranked canonical pathways of *α*-synuclein and *α*-synuclein-associated genes from the dataset. **(D)** Top-ranked diseases and disorders of *α*-synuclein.

IPA predicted 426 canonical signaling pathways for *α*-synuclein. Neuroinflammation (*P* 4.78 e-18) was the most prominent of the predicted signaling pathways for *α*-synuclein followed by Huntington’s disease (*P* 4.34 e^−11^), triggering receptor expressed on myeloid cells 1 (TREM1; *P* 1.28 e^−10^), phagosome maturation (*P* 2.96 e^−11^), and sirtuin signaling pathways (*P* 5.50 e^−10^; Figure 2A). The major canonical signaling pathways of *α*-synuclein are presented in Supplementary Figures S1–S6. Next, we grouped *α*-synuclein and its interacting partner genes from the NCBI dataset that appeared across all the pathways together and performed a frequency distribution analysis. The analysis showed that 168 of 215 NCBI dataset genes appeared 1942 times across all the predicted canonical pathways. The analysis further revealed that nuclear factor-kappaB (NF-κB) protein subunit 1 gene (NFKB1) was the most populated gene (7.6%) across all the pathways followed by Fos proto-oncogene AP-1 transcription factor subunit (FOS; 5.1%), protein kinase C delta (PRKCD; 4.9%), tumor necrosis factor (TNF; 4.7%), glycogen synthase kinase 3 beta (GSK3B; 4.0%), cell division control protein 42 homolog (CDC42; 3.7%), interleukin 6 (IL6; 3.1%), mammalian target of rapamycin (MTOR; 3.1%), interleukin 1 beta (IL1B; 2.9%), and interferon-gamma (IFNG; 2.9%; Figure 2B; Supplementary Table S2). Thereafter, we overlapped the major canonical signaling pathways and the NCBI dataset genes expressed in those pathways, which divulged that neuroinflammation was the highest overlapped pathway with the molecules displaying a greater degree of overlapping with TREM1 and sirtuin signaling pathways. Huntington’s disease displayed a relatively higher extent of overlapping with phagosome maturation signaling molecules while exhibiting the least overlapping with TREM1 signaling molecules. Furthermore, among the overlapped molecules, the molecules associated with inflammation, namely NFKB1, TNF, and caspase-1 (CASP1) displayed the highest prevalence across the overlapped pathways (Figure 2C). In our analysis of the pathological role of *α*-synuclein, it was highest implicated in neurological diseases (*P* 2.34 e^−57^), followed by organismal injury and abnormalities (*P* 6.21 e^−56^), skeletal and muscular disorders (*P* 4.55 e^−53^), psychological disorders (*P* 1.96 e^−47^), and hereditary disorders (*P* 6.27 e^−43^; Figure 2D; Supplementary Figure S7).

#### Molecule activity predictor (MAP) analysis of the principal SNCA interaction network

**Figure 3:**
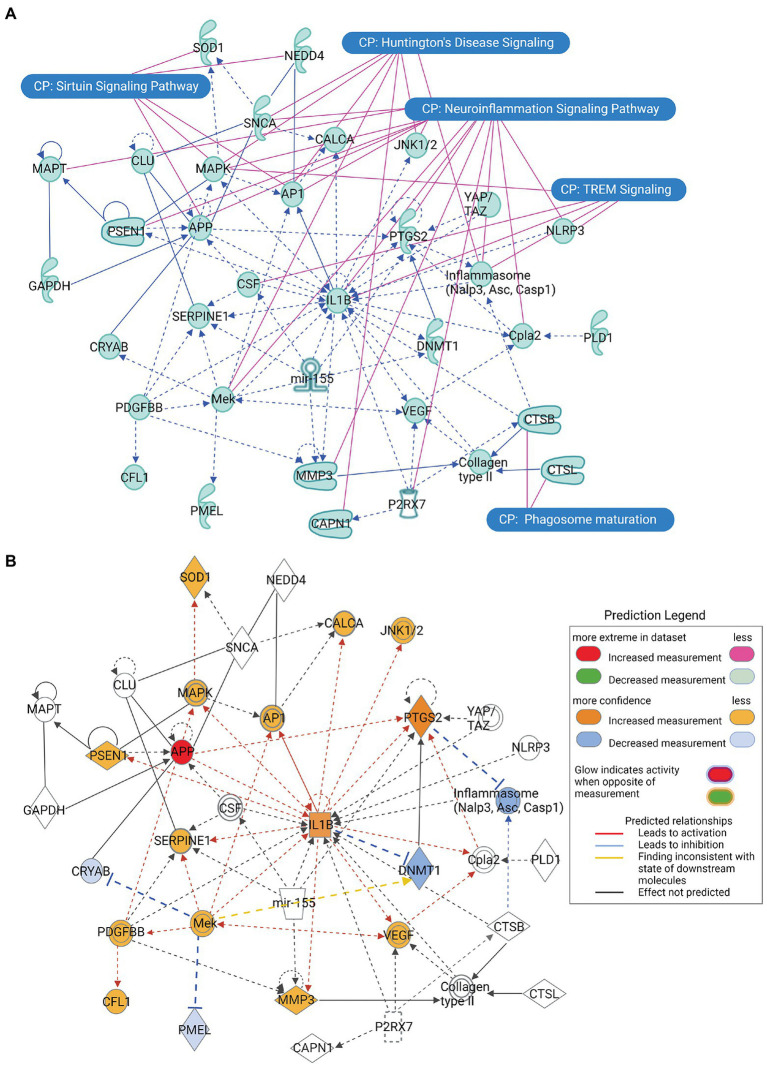
The principal interaction network of *α*-synuclein gene SNCA across all the predicted canonical signaling pathways. **(A)** The principal interaction network of SNCA revealed that SNCA directly interacts with APP, CLU, and NEDD4, whereas it interacts indirectly with CALCA and SOD1. Furthermore, the overlapping of the principal interaction network of *α*-synuclein and top-ranked canonical signaling pathways highlights a relationship between the interaction network genes and canonical pathways. **(B)** The molecule activity predictor (MAP) analysis of the principal interaction network of SNCA, depicting that increase in the activity of APP leads to a series of activation and inhibition of network genes. Figure created with BioRender.com.

The principal interaction network of the *α*-synuclein gene (SNCA) predicted that besides SNCA playing a key role in neuroinflammation and Huntington’s disease, displayed direct interactions with amyloid beta precursor protein (APP), clusterin (CLU), and neural precursor cell expressed developmentally down-regulated protein 4 (also called NEDD4 E3 ubiquitin-protein ligase; NEDD4) genes, whereas it showed indirect relationships with calcitonin related polypeptide alpha (CALCA) and superoxide dismutase 1 (SOD1) genes (Figure 3A). Our attempt to predict the direct effect of the activation or inhibition of SNCA on the other genes of the principal interactome was not met with success. However, we observed that the increased activity of APP leads to the activation of pro-inflammatory genes IL1B and prostaglandin-endoperoxide synthase 2 (PTGS2; also known as cyclooxygenase-2 or COX-2) genes, which, in turn, activates the genes exhibiting the direct relationship with SNCA, namely CALCA and SOD1 (Figure 3B).

#### Prediction of upstream SNCA regulators

Along with the canonical pathways, interaction network, diseases, and biological functions, the core expression analysis of the *α*-synuclein NCBI gene dataset also predicted the upstream regulators of SNCA (Table 1). IL1B was the highest-ranked upstream regulator of SNCA followed by APP, TNF, macrophage migration inhibitory factor (MIF), and mitogen-activated protein kinase kinase 1 (MEK). Observation of the predicted top-thirty upstream regulators indicated that these molecules were associated with the protein aggregation (APP), inflammation and innate immunity (IL1B, TNF, MIF, PTGS2, CCL2, CD14, IL18, TLR4, CD163, IL27, lymphotoxin, IL33, NFKB, ADIPOQ, IGNG, STAT3, IgG, C5, and P2RX7), and neuronal cell growth, differentiation, and transcription regulation (Mek, ERK, P38 MAPK, MAP3K7, and HIF1A). Besides, molecules concerning the regulation of synaptic receptors (ARRB2), neurotransmission (TAC1), and post-transcriptional regulations (mir-155 and miR-155-5p) also appeared in the list of prominent upstream SNCA regulators.

**Table 1:** The upstream regulators of *α*-synuclein (SNCA) predicted by expression analysis of the *α*-synuclein NCBI gene dataset using IPA.

Rank	Upstream regulator	Molecule type	*P*-value of overlap
1	IL1B	Cytokine	1.52 e^−10^
2	APP	Other	1.81 e^−10^
3	TNF	Cytokine	3.47 e^−10^
4	MIF*	Cytokine	7.03 e^−10^
5	Mek* (MAPK/ERK kinase 1)	Group	9.51 e^−10^
6	ERK* (MAPK)	Group	1.04 e^−09^
7	PTGS2 (COX2)	Enzyme	1.05 e^−09^
8	TP53*	Transcription regulator	1.64 e^−09^
9	P38 MAPK* (MAPK 14)	Group	1.97 e^−09^
10	CCL2	Cytokine	2.17 e^−09^
11	HIF1A*	Transcription regulator	3.87 e^−09^
12	mir-155*	microRNA	4.17 e^−09^
13	CD14*	Transmembrane receptor	7.55 e^−09^
14	IL18	Cytokine	7.89 e^−09^
15	TLR4	Transmembrane receptor	9.20 e^−09^
16	CD163*	Transmembrane receptor	1.04 e^−08^
17	IL27*	Cytokine	1.87 e^−08^
18	Lymphotoxin* (TNFB)	Complex	4.55 e^−08^
19	IL33*	Cytokine	6.69 e^−08^
20	ARRB2*	Other	1.53 e^−07^
21	TAC1*	Other	1.53 e^−07^
22	NFKB	Complex	1.84 e^−07^
23	MAP3K7	Kinase	1.92 e^−07^
24	miR-155-5p* (miRNAs w/seed UAAUGCU)	Mature microRNA	2.00 e^−07^
25	ADIPOQ* (Adiponectin C1Q)	Other	2.00 e^−07^
26	IFNG	Cytokine	2.40 e^−07^
27	STAT3*	Transcription regulator	2.58 e^−07^
28	IgG*	Complex	2.83 e^−07^
29	C5	Cytokine	2.86 e^−07^
30	P2RX7	Ion channel	2.86 e^−07^
31	COL18A1*	Other	3.45 e^−07^
32	LILRB4*	Other	3.55 e^−07^
33	ATF3	Transcription regulator	4.13 e^−07^
34	CD40LG	Cytokine	6.07 e^−07^
35	PI3K* (family)	Group	6.12 e^−07^
36	IL1A	Cytokine	6.12 e^−07^
37	DDX58*	Enzyme	7.96 e^−07^
38	ABCA1	Transporter	1.04 e^−06^
39	IL32	Cytokine	1.07 e^−06^
40	Creb	Group	1.09 e^−06^
41	TNFSF12*	Cytokine	1.26 e^−06^
42	IL37	Cytokine	1.26 e^−06^
43	Akt* (PKB)	Group	1.36 e^−06^
44	CSF*	Group	1.36 e^−06^
45	NAMPT	Cytokine	2.08 e^−06^
46	PLG*	Peptidase	3.24 e^−06^
47	BMP7*	Growth factor	3.46 e^−06^
48	JUN	Transcription regulator	3.48 e^−06^
49	CD44*	Other	3.76 e^−06^
50	CASP1*	Peptidase	4.11 e^−06^

*The predictions likely originated from the data obtained from cell culture studies. ABCA1; ATP binding cassette subfamily A member 1, ADIPOQ; Adiponectin C1Q, Akt; Serine/Threonine kinase 1/PKB, ARRB2; Arrestin beta-2, ATF3; Activating transcription factor 3, BMP7; Bone morphogenetic protein 7, C5; Complement component 5, CCL2; C-C motif chemokine ligand 2, CD14/163/40/44; Cluster of differentiation 14/163/40/44, CD40LG; CD40 ligand, COL18A1; Collagen type XVIII alpha 1 chain, Creb; cAMP-response element binding protein, CSF; Colony stimulating factor, DDX58; DExD/H-box helicase 58, ERK; Extracellular signal-regulated kinase/MAPK, HIF1A; Hypoxia inducible factor 1 subunit alpha, IgG; Immunoglobulin G, JUN; Jun proto-oncogene/AP1, LILRB4; Leukocyte immunoglobulin like receptor B4, Mek; Mitogen-activated protein kinase kinase 1/MAPK/ERK kinase 1, MIF; Macrophage migration inhibitory factor, mir-155 (miRNAs w/seed UAAUGCU); microRNA 155, NAMPT; Nicotinamide phosphoribosyltransferase, P2RX7; Purinergic receptor P2X 7, P38 MAPK; MAPK P38 alpha/MAPK 14, PI3K; Phosphoinositide 3-kinases, PLG; Plasminogen, STAT3; Signal transducer and activator of transcription 3, TAC1; Tachykinin precursor 1, TP53; Tumor protein P53.

### IPA toxicity analysis of *α*-synuclein, amyloid beta, tau, and Huntingtin (HTT)

#### Prediction of *α*-synuclein, amyloid beta, tau, and Huntingtin toxicity signaling pathways

**Figure 4:**
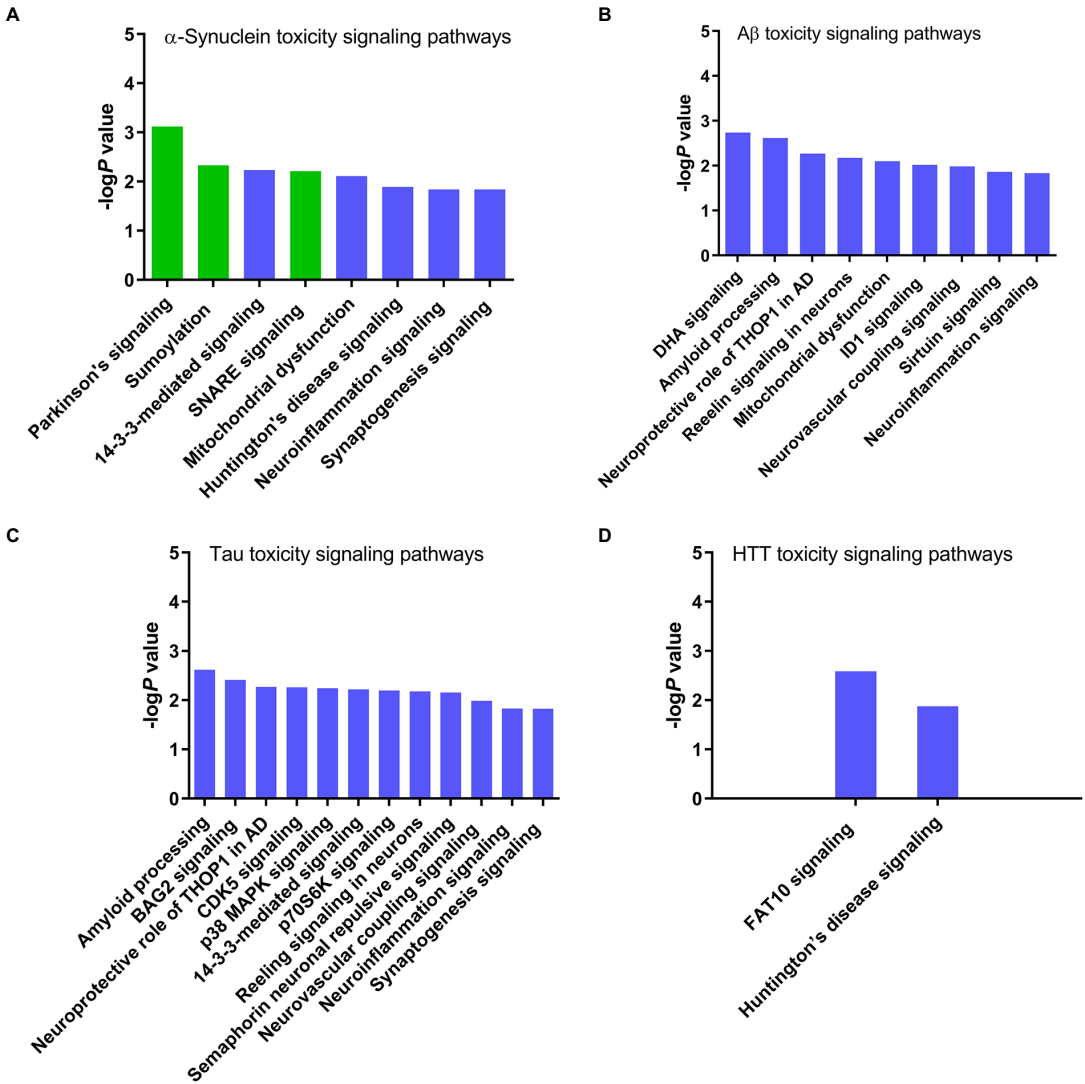
Canonical signaling pathways associated with the toxicity of neurodegenerative proteins predicted by IPA. **(A)** *α*-Synuclein, **(B)** Amyloid beta, **(C)** Tau, and **(D)** Huntingtin. IPA toxicity analysis identified Parkinson’s, sumoylation, and SNARE signaling pathways specific to the *α*-synuclein toxicity. Conversely, *α*-synuclein shares 14–3-3, mitochondrial dysfunction, Huntington’s disease, neuroinflammation, and synaptogenesis signaling pathways with other neurotoxic proteins amyloid beta, tau, and HTT, suggesting that these pathways are likely activated due to the host cell stress response or secondary non-specific response to the toxicity of *α*-synuclein.

**Figure 5:**
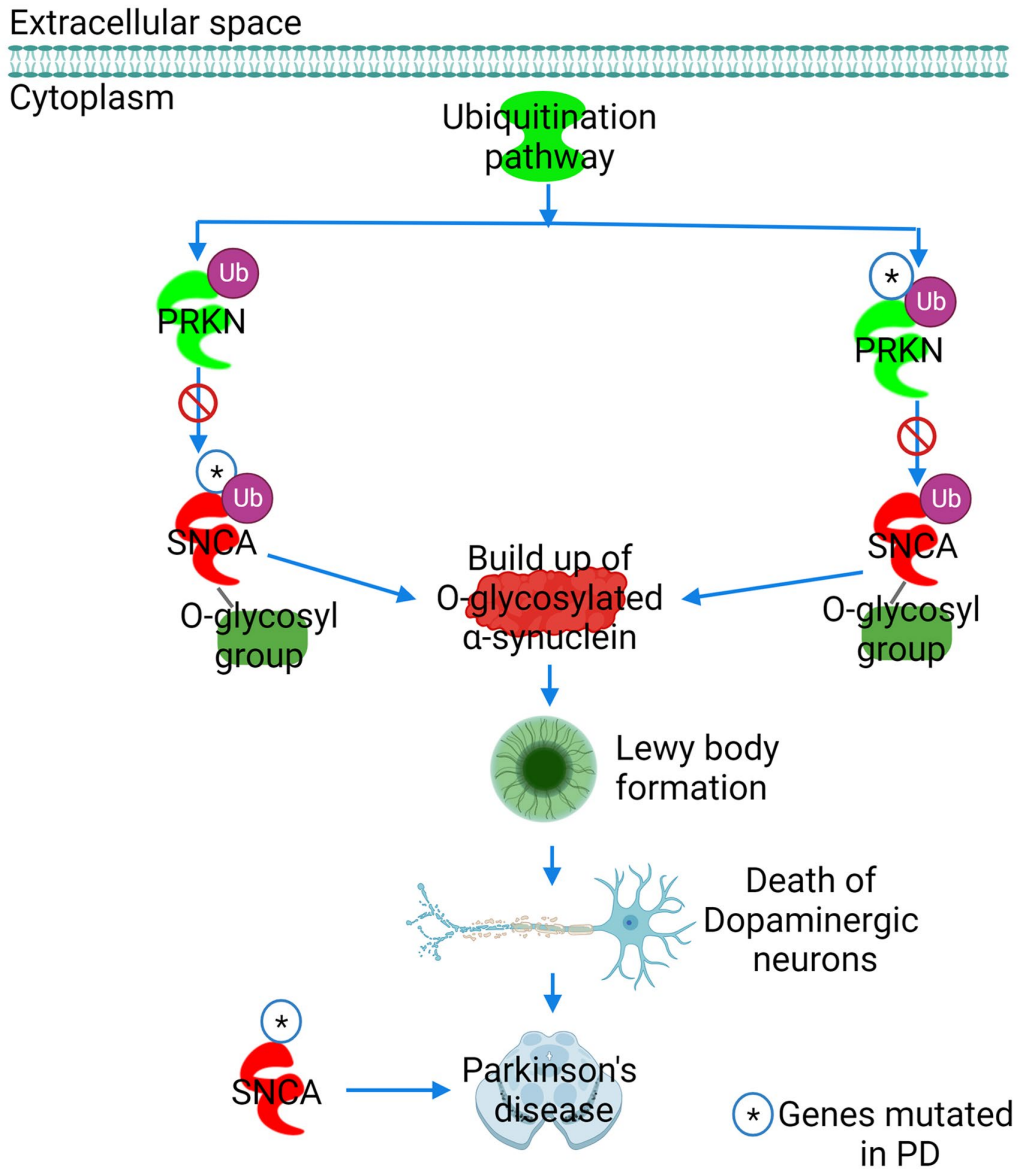
The role of *α*-synuclein in Parkinson’s disease (PD) predicted by IPA. PD was ranked as the leading pathway of *α*-synuclein toxicity. The E3 ubiquitin ligase Parkin (PRKN) labels the glycosylated form of synuclein (SNCA) for proteasomal degradation. Mutation in either PRKN or SNCA precludes PRKN from binding to SNCA, preventing the proteasomal degradation of glycosylated *α*-synuclein. A build-up of glycosylated *α*-synuclein in neuronal cells results in the formation of Lewy bodies, leading to dopaminergic neuron death and PD. Figure created with BioRender.com.

The core expression analysis of SNCA predicted canonical signaling pathways, upstream regulators, and interaction network molecules belonging to both physiological functions and toxicity of *α*-synuclein. Therefore, to identify the pathways and network molecules specific to *α*-synuclein toxicity, we performed the toxicity analysis of *α*-synuclein. IPA toxicity analysis of SNCA predicted PD signaling (*P* 7.50 e^−04^), sumoylation (*P* 4.73 e^−03^), 14–3-3 (*P* 5.91 e^−03^), SNARE (*P* 6.19 e^−03^), mitochondrial dysfunction (*P* 7.74 e^−03^), Huntington’s disease (*P* 1.30 e^−02^), neuroinflammation (*P* 1.44 e^−02^), and synaptogenesis (*P* 1.46 e^−02^) as the canonical signaling pathways of *α*-synuclein toxicity (Figure 4A). The role of *α*-synuclein in PD disease is illustrated in Figure 5 while the rest of the pathways concerning *α*-synuclein toxicity are depicted in Supplementary Figures S2, S3, S8–S12.

After exploring *α*-synuclein toxicity pathways, we also analyzed the toxicity profiles of similar pathogenic proteins, which cause neurodegeneration, such as amyloid beta, tau, and Huntingtin to rule out the *α*-synuclein toxicity pathways arising due to the host cell stress response or secondary non-specific response to the *α*-synuclein toxicity. The toxicity analysis of APP predicted docosahexaenoic acid (DHA) signaling (*P* 1.82 e^−03^), amyloid processing (*P* 2.4 e^−03^), neuroprotective role of thimet oligopeptidase (THOP1) in AD (*P* 5.37 e^−03^), reelin signaling in neurons (*P* 6.62 e^−03^), mitochondrial dysfunction (*P* 7.91 e^−03^), inhibitor of differentiation-1 (ID1 signaling (*P* 9.59 e^−03^), neurovascular coupling signaling (*P* 1.03 e^−02^), sirtuin signaling (*P* 1.37 e^−02^), and neuroinflammation signaling (*P* 1.47 e^−02^) pathways as the mediators of amyloid beta toxicity (Figure 4B). At the same time, the toxicity analysis of MAPT identified amyloid processing (*P* 2.4 e^−03^), Bcl-2-associated athanogene 2 (BAG2) signaling (*P* 3.88 e^−03^), neuroprotective role of THOP1 in AD (*P* 5.37 e^−03^), CDK5 signaling (*P* 5.47 e^−03^), p38 MAPK signaling (*P* 5.75 e^−03^), 14–3-3-mediated signaling (*P* 6.04 e^−03^), p70S6K signaling (*P* 6.38 e^−03^), reelin signaling in neurons (*P* 6.62 e^−03^), semaphorin neuronal repulsive signaling (*P* 6.95 e^−03^), neurovascular coupling signaling (*P* 1.03 e^−02^), neuroinflammation signaling (*P* 1.47 e^−02^), and synaptogenesis signaling (*P* 1.49 e^−02^) pathways as the architects of tau-induced toxicity in neurodegenerative diseases (Figure 4C). We also performed the toxicity analysis of HTT, which predicted human leukocyte antigen F-associated transcript10 (FAT10) signaling (*P* 2.59 e^−03^) and Huntingtin’s disease signaling (*P* 1.33 e^−02^) pathways as the conveners of toxicity caused by Huntingtin’s protein (Figure 4D).

## Discussion

### Prediction of *α*-synuclein canonical pathways

The core expression analysis of the *α*-synuclein NCBI gene dataset predicted 426 canonical pathways. These pathways were ranked based on Fisher’s exact test *P*-values. Neuroinflammation signaling was predicted to be the foremost canonical pathway of *α*-synuclein followed by Huntington’s disease, TREM1 signaling, phagosome maturation, and sirtuin signaling pathways (Figure 2A; Supplementary Figures S2–S6). Abnormal expression of SNCA forms LB in neuronal cells, which activates neuroinflammation signaling genes, like advanced glycosylation end-product specific receptor (AGER), toll-like receptor 2 (TLR2), and toll-like receptor 4 (TLR4). Activation of these genes triggers downstream inflammation marker genes activator protein 1 (AP1) and NFKB, which, in turn, stimulate a cascade of pro-inflammatory genes, like TNF, IL6, IL1B, and IFNG, etc., eventually resulting in neuronal cell death (Supplementary Figure S2). Activation of SNCA in Huntington’s disease conveys an inhibitory effect on DnaJ heat shock protein family (Hsp40) member B1 (DNAJB1). The latter protein prevents the aggregation propensity of misfolded proteins *via* the activation of brain-derived neurotrophic factor (BDNF; Supplementary Figure S3). SNCA mediates innate immunity *via* TREM1 signaling. As the name indicates, TREM1 is expressed on myeloid or blood cells, like monocytes, eosinophils, neutrophils, basophils, and macrophages and its activation *via* inflammatory mediators amplifies immune response. TREM1-induced neuroinflammation is facilitated by common inflammatory mediators, such as NF-κB, TNF, IL6, IL1B, TLR1 and 2, NOD-like receptor pyrin domain-containing protein 3 (NLRP3), and intercellular adhesion molecule 1 (ICAM1; Supplementary Figure S4). *α*-Synuclein plays a vital role in the host defense mechanism, in the event of viral or bacterial infections (Alam et al., 2022). SNCA augments innate immunity through an interaction with Ras-related GTPases Rab5 and Rab7, cathepsins, and lysosomal-associated membrane protein 2 (LAMP2), which activates human leukocyte antigen—DR isotype (HLA-DR) or MHC class II cell surface receptor, resulting into phagolysosome of pathogens. During phagosome maturation, Rab5 interacts with SNARE proteins and vacuolar(H^+^)-ATPase (V-ATPase) to impetus the maturation of the early endosome, whereas Rab7 is essential for the fusion of phagosome with the lysosome. V-ATPase acidifies phagosomes by translocation of H^+^ ions, resulting in the suppression of microbial growth (Supplementary Figure S5). Acetylation is a key regulatory mechanism for the aggregation of *α*-synuclein. Sirtuins (SIRT) are histone deacetylases, which play an important role in inflammation, aging, and cancer (Supplementary Figure S6). Sirtuins, namely SIRT2, which is predominantly localized in the cytoplasm deacetylates *α*-synuclein, resulting in exacerbated *α*-synuclein toxicity and neurodegeneration. (Outeiro et al., 2007; de Oliveira et al., 2017). Taken together, SNCA exercises its role in inflammation and immune response through inflammatory mediators *via* neuroinflammation and TREM1 signaling pathways and through cathepsin proteases *via* phagosome maturation pathways. Over-activation of SNCA exacerbates Huntingtin protein (HTT)-induced toxicity in Huntington’s disease, while its deacetylation by sirtuins affects its aggregation propensity.

### Analysis of SNCA-interacting NCBI dataset genes expressed in the predicted canonical pathways of *α*-synuclein

Ingenuity pathway expression analysis of the *α*-synuclein NCBI gene dataset predicted 426 canonical pathways for *α*-synuclein. A frequency distribution analysis of the *α*-synuclein dataset genes that appeared in the predicted canonical pathways revealed that 168 genes from the dataset of 215 genes appeared 1942 times across the predicted pathways. NFKB was the most frequently occurring interaction partner of SNCA with a prevalence of 7.6%. NFKB gene was followed by FOS (5.1%), PRKCD (4.9%), TNF (4.7%), GSK3B (4.0%), CDC42 (3.7%), IL6 (3.1%), MTOR (3.1%), IL1B (2.9%), and IFNG (2.9%). The major interaction partners of SNCA are presented in Figure 2B. The predominant presence of transcription factors NFKB and FOS, cytokines TNF, IFNG, IL-6, and IL1B, cell cycle regulator CDC42, cell growth, differentiation, and motility regulating kinases PRKCD and MTOR, and cytokine regulating kinase GSK3B in the SNCA interactome indicates that SNCA gene is an important mediator of neuroinflammation and neural cell growth and motility. Furthermore, MTOR and GSK3B are also being investigated as the potential targets for the therapeutic intervention of neurodegenerative diseases and aging. The increased MTOR signaling is associated with the build-up of A*β* (Caccamo et al., 2010) and *α*-synuclein plaques (Pérez-Revuelta et al., 2014), whereas GSK3B phosphorylates *α*-synuclein and tau proteins, which leads to accumulation and aggregate formation of these proteins (Credle et al., 2015). MTOR- and GSK3B-mediated increased *α*-synuclein activity modulates the level of CDC42, which affects neuronal outgrowth (Schnack et al., 2008). At the same time, *α*-synuclein aggregates upregulate PRKCD activity, leading to mitochondrial dysfunction, endoplasmic reticulum stress, and activation of inflammatory cascade mediated by NFKB, TNF, IL-6, and IL1B, etc. (Samidurai et al., 2021). In summary, molecules like MTOR and GSK3B induce *α*-synuclein aggregation, which, in turn, dysregulates the activity of PRKCD and CDC42, resulting in perturbed neuronal architecture and neuroinflammation.

### Overlapping of the top-ranked canonical pathways and the *α*-synuclein NCBI gene dataset

Overlapping of IPA-predicted top canonical pathways and *α*-synuclein-associated genes could predict the common features of these pathways as well as reveal the otherwise unobserved molecular patterns of these pathways. Overlapping of the top-ranked canonical pathways and the *α*-synuclein NCBI gene dataset (Figure 2C) predicted that neuroinflammation signaling was the most overlapped pathway with 15 molecules displaying connections with the other pathways. Furthermore, neuroinflammation signaling shared the highest number of molecules with TREM1 signaling followed by sirtuin signaling pathways, indicating a plethora of cross-talks across these pathways. The presence of molecules, like ICAM1, TLR1/2, NLRP3, IL1B, NFKB1, and HLA-DRB1, and myeloid differentiation primary response 88 (MYD88) in both neuroinflammation and TREM1 signaling suggests the overwhelming role of neuroinflammation and immune response in TREM1 signaling. On the other hand, the occurrence of APP, GSK3B, nuclear factor (erythroid-derived 2)-like 2 (NFE2L2), TNF, and NFKB1 in both neuroinflammation and sirtuin signaling indicates shared functions of both the pathways in protein aggregation, oxidative stress, and neuroinflammation. Besides, Huntington’s disease signaling molecules displayed the highest overlapping with the phagosome maturation signaling molecules. The presence of SNAP25, VAMP2, and YKT6 (synaptobrevin or V-SNARE homolog) in both the pathways points toward the shared role of both the signaling in exocytosis. As expected, SNCA was a mutual link between Huntington’s disease and neuroinflammation signaling pathways. Coming to the molecules shared by all the overlapping pathways, the molecules related to inflammation and cell death, namely TNF, NFKB1, and CASP1 were markedly shared across the pathways. Taken together, the overlapping of IPA-predicted top-ranked canonical pathways and the *α*-synuclein NCBI gene dataset substantiate that neuroinflammation is the most prominent signaling pathway of *α*-synuclein.

### Prediction of *α*-synuclein-associated diseases

Analysis of diseases and biological functions linked to *α*-synuclein predicted neurological diseases, organismal injury and abnormalities, skeletal and muscular disorders, psychological disorders, and hereditary disorders as the major pathological outcomes of SNCA gene anomalies (Figure 2D; Supplementary Figure S7). Among SNCA-linked diseases and disorders, PD, Alzheimer’s disease (AD), tauopathy, degenerative dementia, progressive motor neuropathy, familial encephalopathy, hereditary and motor neuropathies, a disorder of basal ganglia, movement disorders, neuromuscular diseases, and other progressive neurological diseases were found to be the major diseases and disorders linked to SNCA gene. The recent research on *α*-synuclein associated diseases suggests that PD, DLB, and MSA, where the misfolding of *α*-synuclein monomers into oligomers followed by progressive transformation of oligomers into amyloid fibrils takes place, have become a central point of current synucleinopathy research (Oliveira et al., 2021). As a whole, abnormalities in *α*-synuclein expression primarily cause aggregation of *α*-synuclein monomers into fibrils, which eventually form LB, LN, and glial cell inclusions—the pathological hallmarks of PD, DLB, and MSA.

### MAP analysis of the principal SNCA interaction network

IPA predicted several interaction networks of SNCA and ranked them according to their statistical likelihood of molecules being appeared in the network by random chance. The top-ranked interaction network of SNCA (score 33) revealed that SNCA directly interacts with APP, CLU, and NEDD4, whereas it indirectly interacts with CALCA and SOD1 (Figure 3A). SNCA increases the activity of APP *via β-and γ*-secretase, leading to amyloid plaque formation (Roberts et al., 2017). Astrocytes play a vital role in the clearance of *α*-synuclein aggregates *via* the endolysosomal pathway. CLU directly binds to *α*-synuclein aggregates and limits their internalization into astrocytes; thus, contributing to the pathogenesis of PD (Filippini et al., 2021), whereas NEDD4 ligase ubiquitinates *α*-synuclein and clears *α*-synuclein aggregates *via* endolysosomal pathway; thereby protecting against *α*-synuclein-induced progressive neurodegeneration (Tofaris et al., 2011). CALCA potentiates the aggregation of *α*-synuclein and activates inflammation mediators, whereas inhibition of CALCA by a small molecule attenuates *α*-synuclein aggregation properties (Na et al., 2020). Mutations in SOD1 cause familial amyotrophic lateral sclerosis (ALS). *α*-Synuclein aggregates interact with SOD1 and promote its oligomerization and aggregation (Helferich et al., 2015). Altogether, APP, CLU, CALCA, and SOD1 may contribute to the *α*-synuclein-associated synucleinopathy, whereas NEDD4 by posttranslational modification protects against the formation of toxic *α*-synuclein inclusions.

We also performed the MAP analysis of the principal SNCA network to observe whether the increase or decrease in the activity of SNCA activates or inhibits the interacting genes (Figure 3B). Unfortunately, the MAP analysis of SNCA did not predict any activity pattern; therefore, we attempted MAP analysis of other genes which were directly interacting with SNCA, where the increase in the activity of APP was associated with the exacerbation of IL1B activity, which, in turn, activated a battery of molecules. APP-IL1B axis-mediated activation of catalytic subunit of *γ*-secretase (presenilin-1 or PSEN) breaks down APP, leading to the increased synthesis of A*β*. Proteolytic degradation of APP further activates protein kinases concerning cell death mitogen-activated protein kinase (Mapk) and C-Jun N-terminal kinase (JNK1/2), transcription factor Ap1, inflammatory mediators PTGS2 (COX2) and vascular endothelial growth factor (Vegf), cytosolic phospholipase A2 (Cpla2) for lipid biosynthesis, matrix metallopeptidase 3 (MMP3), innate immunity component serine proteinase (SERPINE1), and calcium regulating CALCA for potentiating aggregate formation, orchestrating a cascade of neuroinflammation and cell death. At the same time, the activation of IL1B by APP leads to the inhibition of DNA methyltransferase 1 (DNMT1), a molecule responsible for maintaining DNA methylation for epigenetic gene regulation. As a whole, the MAP analysis of the principal SNCA interaction network suggests that the aggregate formation of *α*-synuclein accompanies neuroinflammation, inevitably leading to cell death in neurodegenerative diseases.

### Prediction of SNCA-upstream regulators

Besides, the predictions of canonical signaling pathways, interaction networks, and disease and biological functions, the core expression analysis of the *α*-synuclein NCBI gene dataset also predicted upstream regulators of SNCA (Table 1). The presence of APP in the list of revealed upstream regulators indicates that protein aggregation occurs during synucleinopathy. Since protein aggregation formation is always associated with neuroinflammation, the overwhelming presence of cytokines (IL1B, TNF, MIF, CCL2, IL18, IL27, lymphotoxin, IL33, IFNG, IgG, and C5), inflammatory mediator (PTGS2/COX2), transmembrane receptors mediating innate immunity (CD14, TLR4, and CD163), transcription regulators (TP53, HIF1A, NFKB, and STAT3), post-transcriptional gene regulators (mir-155 and miR-155-5p), and purinergic receptor for macrophage lysis (P2RX7) as the top-ranked upstream regulators substantiate that neuroinflammation is a hallmark of synucleinopathy in the brain. In addition, the presence of MEK, ERK, P38 MAPK, and MAP3K7 kinases as the upstream SNCA regulators suggests that stress and inflammatory cytokines induce the expression of MAPK signal transduction pathway members. Activated MAP kinases then collude with SNCA to cause synaptic dysfunction and neurodegeneration. As a whole, the upstream SNCA-regulating molecules underlie neuroinflammation and innate immune response as key events in *α*-synuclein pathology.

### Analysis of SNCA, APP, MAPT, and HTT toxicity signaling pathways

IPA expression analysis of the *α*-synuclein NCBI gene dataset predicted canonical signaling pathways concerning physiological functions, toxicity, and host cell stress response to the toxicity of *α*-synuclein (Figure 2A; Supplementary Figure S1). Since with the expression analysis of SNCA and associated genes, we were not able to discriminate the canonical pathways specific to the physiological functions and toxicity of *α*-synuclein, we performed the toxicity analysis of SNCA to reveal the canonical signaling pathways specific to *α*-synuclein toxicity. The toxicity analysis of SNCA identified PD followed by sumoylation, 14–3-3, SNARE, mitochondrial dysfunction, Huntington’s disease, neuroinflammation, and synaptogenesis as the canonical signaling pathways of *α*-synuclein-associated toxicity (Figure 4A). Here, we remind that the expression analysis of the *α*-synuclein NCBI gene dataset had predicted neuroinflammation, Huntington’s disease, TREM1, phagosome maturation, and sirtuin signaling as the major canonical pathways of *α*-synuclein (Figure 2A). As we can observe upon comparing the expression analysis- and toxicity analysis-predicted pathways that the top-ranked pathways of expression analysis, neuroinflammation and Huntingtin’s disease also appeared in the list of pathways predicted by toxicity analysis of *α*-synuclein, suggesting that these pathways are likely associated with the toxicity of *α*-synuclein (Figures 2A, 4A). On the contrary, the absence of other top-ranked expression analysis-predicted pathways, such as TREM1, phagosome maturation, and sirtuin signaling in the list of pathways predicted through the toxicity analysis of *α*-synuclein, indicates that these pathways are more likely related to the physiological functions of *α*-synuclein than its toxicity. Thus, the comparison of the expression analysis-predicted pathways with the toxicity analysis-predicted pathways points out that the top-ranked pathways of expression analysis—neuroinflammation and Huntingtin’s disease are likely associated with the abnormal expression of *α*-synuclein, whereas the rest of the three pathways such as TREM1, phagosome maturation, and sirtuin signaling pathways are likely related to the normal physiological functions of *α*-synuclein.

To gain further insight into the IPA-predicted toxicity pathways, and whether any of the predicted pathways is a result of the host cell stress response instead of *α*-synuclein pathogenicity, we compared the toxicity pathways of *α*-synuclein (Figure 4A) with that of other neurodegenerative proteins, such as amyloid beta (Figure 4B), tau (Figure 4C), and Huntingtin (Figure 4D). These proteins form toxic aggregates in a similar fashion to *α*-synuclein. IPA toxicity analysis of these proteins revealed that mitochondrial dysfunction and neuroinflammation signaling pathways were common to both *α*-synuclein and amyloid beta while 14–3-3, neuroinflammation, and synaptogenesis signaling pathways were common between *α*-synuclein and tau. Furthermore, the toxicity analysis of HTT identified Huntingtin’s disease signaling as one of the major pathways and a common link between Huntingtin’s protein and *α*-synuclein. Thus, the appearance of 14–3-3, mitochondrial dysfunction, Huntington’s disease, neuroinflammation, and synaptogenesis signaling as the common toxicity pathways for *α*-synuclein and other neurotoxic proteins amyloid beta, tau, and Huntingtin suggest that these pathways are likely activated as a result of the host cell stress response or secondary non-specific response to the toxicity and immunogenicity of *α*-synuclein rather than as a direct consequence of the *α*-synuclein toxicity. Conversely, the revelation of PD, sumoylation, and SNARE signaling pathways exclusive to the toxicity of *α*-synuclein suggests that these pathways are likely an outcome of direct *α*-synuclein pathogenicity.

The appearance of the PD signaling pathway as the chief architect of *α*-synuclein-induced toxicity was expected as the mutation in the SNCA gene has been reported to cause familial PD (Figures 4A, 5). In normal cellular conditions, the glycosylated form of *α*-synuclein is a substrate for Parkin (PARK2), an E3 ubiquitin ligase, which marks *α*-synuclein for proteasomal degradation (Shimura et al., 2001). IPA predicted that due to mutations in either Parkin or *α*-synuclein gene (SNCA), Parkin fails to bind to the glycosylated *α*-synuclein. *α*-Synuclein in the glycosylated state then accumulates in the neuronal cells, forming LB, which causes the death of dopaminergic neurons and the progression of PD (Figure 5). IPA divulged that the sumoylation mechanism was the second-leading contributor to the *α*-synuclein-induced toxicity (Figure 4A; Supplementary Figure S8). Sumoylation is a posttranslational modification required for normal cellular functions, where a small ubiquitin-like modifier (SUMO) protein covalently binds to the target protein to perform mundane activities. Our study predicted that the sumoylation of *α*-synuclein by SUMO-conjugating E2 enzyme UBC9 (UBE2I) and E3 SUMO-protein ligase chromobox 4 (CBX4) promotes *α*-synuclein aggregation (Supplementary Figure S8). However, our finding that the sumoylation causes *α*-synuclein aggregation should be taken with caution because the fate of *α*-synuclein sumoylation depends upon the isoform and type of SUMO involved in the modification of *α*-synuclein, and many other studies have reported contrasting results. SNARE signaling was another pathway found to be explicit for *α*-synuclein toxicity and function (Figure 4A; Supplementary Figure S10). *α*-Synuclein is a presynaptic protein, which during the SNARE signaling binds to the synaptic vesicle-associated membrane protein synaptobrevin (VAMP2) and forms a SNARE complex in coordination with another synaptic vesicle protein synaptotagmin and the presynaptic plasma membrane proteins syntaxin-1 and SNAP25. The formation of the SNARE complex eventually leads to the exocytosis of synaptic vesicles, also referred to as the release of neurotransmitters (Supplementary Figure S10). Toxic α-synuclein inclusions disrupt the integrity of the synaptic vesicle membrane, leading to neurotoxicity.

### Limitations of the study

The IPA predictions are affected by the gene dataset used for analysis. In due course, updates in NCBI dataset genes concerning *α*-synuclein may affect the IPA prediction about the biological roles and functions of *α*-synuclein. Furthermore, IPA predictions are derived from a dataset of context-dependent experimental outcomes, available on the IPA server. The future update in the IPA dataset or opting for different experimental contexts, either during the Bioinformatics predictions or while replicating the IPA predictions in the wet lab, like opting for different cells, tissues, organs, species, and experimental conditions, may diverge the IPA predictions. Furthermore, IPA predictions are derived from various data sources, including the data originating from RNA sequencing studies. IPA cannot classify if the *α*-synuclein used as an external reagent in some of those RNA sequencing studies was endotoxin contaminated, which might have affected IPA predictions. Besides, three different species of *α*-synuclein, oligomers, protofibrils, and amyloid fibrils, confer different levels of toxicity. The current version of the IPA cannot identify the species responsible for the predicted toxic effect.

## Conclusion

To our knowledge, this work is the first report on the analysis of signaling pathways, network molecules, biological functions, and role of *α*-synuclein using the Bioinformatics tool, IPA. The expression and toxicity analyzes of *α*-synuclein identified TREM1, phagosome maturation, and sirtuin signaling pathways associated with the physiological functions of *α*-synuclein, whereas PD, sumoylation, and SNARE signaling pathways were identified as specific to the toxicity of *α*-synuclein. From the NCBI gene dataset used for IPA predictions of *α*-synuclein, NFKB1, FOS, PRKCD, TNF, GSK3B, CDC42, IL6, MTOR, PLCB1, and IL1B were the highest populated genes across the predicted canonical signaling pathways. According to the overlap of the top-ranked canonical pathways, neuroinflammation signaling was the most overlapped pathway while inflammatory mediators NFKB1, TNF, and CASP1 were the most shared molecules across the overlapped pathways. Neurological diseases were predicted to be the top pathological conditions associated with *α*-synuclein. A MAP analysis of the principal interaction network of SNCA revealed a direct relationship between SNCA and APP, suggesting that collusion between SNCA and APP likely produces neurotoxic effects. Besides, IPA predicted IL1B, APP, TNF, MIF, Mek, ERK, PTGS2, TP53, M38 MAPK, and CCL2 as the top upstream regulators of SNCA. The appearance of APP, a series of inflammatory mediators, and MAPK kinases as the upstream regulators indicate that the upstream SNCA regulators contribute to synucleinopathy. On the whole, IPA expression analysis of *α*-synuclein and associated genes reveals that *α*-synuclein is a key conspirator in protein aggregopathy and neuroinflammation, where the abnormal expression of *α*-synuclein activates cerebral amyloid peptide APP, a battery of cytokines, like NFKB1, TNF, IL1B, etc., and MAPK family kinases, like Mek, ERK, and P38 MAPK. Besides, our study provides an important piece of information for wet-lab researchers interested in further exploring signaling, molecular interaction networks, and biological functions of *α*-synuclein.

## Data availability statement

The datasets presented in this study can be found in online repositories. The names of the repository/repositories and accession number(s) can be found in the article/Supplementary material.

## Author contributions

SS conceived the idea, performed the analysis, and wrote and reviewed the manuscript. S-YL conceived the idea, provided suggestions, and reviewed the manuscript. All authors contributed to the article and approved the submitted version.

## Funding

This work was supported by a Brain Pool research grant no. 2021H1D3A2A02044867 from the National Research Foundation (NRF) of Korea, and a research grant no. GCU-2018-0703 from the Gachon University.

## Conflict of interest

The authors declare that the research was conducted in the absence of any commercial or financial relationships that could be construed as a potential conflict of interest.

## Publisher’s note

All claims expressed in this article are solely those of the authors and do not necessarily represent those of their affiliated organizations, or those of the publisher, the editors and the reviewers. Any product that may be evaluated in this article, or claim that may be made by its manufacturer, is not guaranteed or endorsed by the publisher.
