# Supplementary material for: Ingenuity pathway analysis of α-synuclein predicts potential signaling pathways, network molecules, biological functions, and its role in neurological diseases
Source: Front Mol Neurosci. 2022 Nov 29;15:1029682. doi: 10.3389/fnmol.2022.1029682 (PMC9744789; doi:10.3389/fnmol.2022.1029682)
Supplement: Supplementary file 1 [file Data_Sheet_1.PDF]

SUPPLEMENTARY MATERIAL

Ingenuity pathway analysis of *α*-synuclein predicts potential signaling pathways, network molecules, biological functions, and its role in neurological diseases

Sharad Kumar Suthar[1], Sang-Yoon Lee[1,2]*

[1]Neuroscience Research Institute, Gachon University, Incheon 20565, South Korea

[2]Department of Neuroscience, College of Medicine, Gachon University, Incheon 21936, South Korea

*Corresponding author: Prof. Dr. Sang-Yoon Lee, Tel.: +82-32-822-5360, E-mail: rchemist@gachon.ac.kr

Table of contents

Content	Page
Table S1. The NCBI dataset of α-synuclein and associated genes used for Ingenuity pathway analysis.	S2–S3
Table S2. The percent distribution of SNCA interactome genes across the predicted canonical pathways	S4
Figure S1. The major canonical signaling pathways of α-synuclein.	S5
Figure S2. The role of α-synuclein in the neuroinflammation signaling pathway.	S6
Figure S3. The role of α-synuclein in Huntington's disease.	S7
Figure S4. TREM1 signaling pathway of α-synuclein.	S8
Figure S5. Phagosome maturation signaling pathway of α-synuclein.	S9
Figure S6. Sirtuin signaling pathway of α-synuclein.	S10
Figure S7. The heat map diagram exhibiting diseases and biological functions of α-synuclein.	S11
Figure S8. The role of α-synuclein in the sumoylation signaling pathway.	S12
Figure S9. The role of α-synuclein in the 14-3-3-mediated signaling pathway.	S13
Figure S10. The role of α-synuclein in the SNARE signaling pathway.	S14
Figure S11. The role of α-synuclein in the mitochondrial dysfunction signaling pathway.	S15
Figure S12. The role of α-synuclein in synaptogenesis.	S16

Table S1. The NCBI dataset of α-synuclein and associated genes used for Ingenuity pathway analysis.

No.	Gene ID	Name	No.	Gene ID	Name	No.	Gene ID	Name
1	6622	SNCA	73	7534	YWHAZ	145	10376	TUBA1B
2	348	APOE	74	2934	GSN	146	9101	USP8
3	351	APP	75	3297	HSF1	147	2059	EPS8
4	3569	IL6	76	2641	GCG	148	55740	ENAH
5	4137	MAPT	77	10013	HDAC6	149	950	SCARB2
6	7124	TNF	78	22933	SIRT2	150	6490	PMEL
7	26065	LSM14A	79	7345	UCHL1	151	5861	RAB1A
8	4790	NFKB1	80	1508	CTSB	152	6844	VAMP2
9	3479	IGF1	81	10273	STUB1	153	10458	BAIAP2
10	2099	ESR1	82	4976	OPA1	154	2581	GALC
11	120892	LRRK2	83	1072	CFL1	155	1052	CEBPD
12	207	AKT1	84	10059	DNM1L	156	7402	UTRN
13	3123	HLA-DRB1	85	5653	KLK6	157	375790	AGRN
14	4780	NFE2L2	86	10062	NR1H3	158	6711	SPTBN1
15	6531	SLC6A3	87	10155	TRIM28	159	5025	P2RX4
16	3553	IL1B	88	5515	PPP2CA	160	6478	SIAH2
17	5071	PRKN	89	6477	SIAH1	161	6993	DYNLT1
18	3162	HMOX1	90	9627	SNCAIP	162	4860	PNP
19	2475	MTOR	91	834	CASP1	163	3798	KIF5A
20	7097	TLR2	92	409	ARRB2	164	8535	CBX4
21	3458	IFNG	93	6623	SNCG	165	5641	LGMN
22	3146	HMGB1	94	7054	TH	166	4900	NRGN
23	4313	MMP2	95	488	ATP2A2	167	5414	SEPTIN4
24	3383	ICAM1	96	410	ARSA	168	29904	EEF2K
25	154	ADRB2	97	823	CAPN1	169	11124	FAF1
26	2629	GBA	98	1514	CTSL	170	8975	USP13
27	5054	SERPINE1	99	5354	PLP1	171	25978	CHMP2B
28	6647	SOD1	100	7416	VDAC1	172	51142	CHCHD2
29	23435	TARDBP	101	7299	TYR	173	27101	CACYBP
30	3958	LGALS3	102	1513	CTSK	174	793	HLA1
31	2146	EZH2	103	6556	SLC11A1	175	2580	GAK
32	11315	PARK7	104	406950	MIR16-1	176	6853	SYN1
33	2100	ESR2	105	1901	S1PR1	177	55626	AMBRA1
34	4314	MMP3	106	6616	SNAP25	178	116986	AGAP2
35	3308	HSPA4	107	10133	OPTN	179	10724	OGA
36	2932	GSK3B	108	10628	TXNIP	180	5864	RAB3A
37	335	APOA1	109	1137	CHRNA4	181	4669	NAGLU
38	7341	SUMO1	110	2810	SFN	182	6767	ST13
39	836	CASP3	111	27035	NOX1	183	8525	DGKZ
40	5621	PRNP	112	8766	RAB11A	184	26281	FGF20
41	3320	HSP90AA1	113	1859	DYRK1A	185	2021	ENDOG
42	3064	HTT	114	1460	CSNK2B	186	5874	RAB27B
43	114548	NLRP3	115	7096	TLR1	187	9048	ARTN
44	1410	CRYAB	116	10769	PLK2	188	5481	PPID
45	7052	TGM2	117	7942	TFEB	189	9628	RGS6
46	5663	PSEN1	118	1759	DNM1	190	9672	SDC3
47	19	ABCA1	119	7314	UBB	191	406944	MIR153-1
48	406947	MIR155	120	3060	HCRT	192	3267	AGFG1
49	7276	TTR	121	23236	PLCB1	193	29058	TMEM230
50	7018	TF	122	6620	SNCB	194	23317	DNAJC13
51	8878	SQSTM1	123	9217	VAPB	195	6572	SLC18A3
52	25	ABL1	124	407043	MIR7-1	196	51667	NUB1
53	4353	MPO	125	5337	PLD1	197	116442	RAB39B
54	4170	MCL1	126	23400	ATP13A2	198	9529	BAG5

55	3627	CXCL10	127	5660	PSAP	199	4514	MT-CO3
56	2309	FOXO3	128	7376	NR1H2	200	8224	SYN3
57	1509	CTSD	129	10298	PAK4	201	1608	DGKG
58	1786	DNMT1	130	2280	FKBP1A	202	22895	RPH3A
59	1191	CLU	131	407037	MIR320A	203	1662	DDX10
60	3315	HSPB1	132	11076	TPPP	204	10652	YKT6
61	796	CALCA	133	10131	TRAP1	205	441549	CDNF
62	3375	IAPP	134	8239	USP9X	206	27072	VPS41
63	5580	PRKCD	135	9201	DCLK1	207	8935	SKAP2
64	2597	GAPDH	136	4891	SLC11A2	208	57552	NCEH1
65	65018	PINK1	137	6750	SST	209	93664	CADPS2
66	998	CDC42	138	6950	TCP1	210	3422	IDI1
67	5027	P2RX7	139	80208	SPG11	211	84286	TMEM175
68	4615	MYD88	140	6571	SLC18A2	212	25897	RNF19A
69	135	ADORA2A	141	23642	SNHG1	213	2630	GBAP1
70	4734	NEDD4	142	1141	CHRNB2	214	91734	IDI2
71	3920	LAMP2	143	406949	MIR15B	215	391634	HSP90AB2P
72	4929	NR4A2	144	3902	LAG3			

Taxonomy ID: 9606 for all genes

Table S2. The percent distribution of SNCA interactome genes across the predicted canonical pathways

No.	Gene	%	No.	Gene	%	No.	Gene	%	No.	Gene	%
1	NFKB1	7.62	43	SERPINE1	0.57	85	DNM1L	0.21	127	SIAH1	0.10
2	FOS	5.10	44	HSPB1	0.51	86	EZH2	0.21	128	SLC11A2	0.10
3	PRKCD	4.89	45	KNG1	0.51	87	GAPDH	0.21	129	SLC18A3	0.10
4	TNF	4.74	46	PNP	0.51	88	H2AX	0.21	130	TRAP1	0.10
5	GSK3B	3.96	47	TLR1	0.51	89	HSF1	0.21	131	TRIM28	0.10
6	CDC42	3.71	48	TUBA1B	0.51	90	IDI1	0.21	132	UCHL1	0.10
7	IL6	3.14	49	UBB	0.51	91	IDI2	0.21	133	USP9X	0.10
8	MTOR	3.14	50	BAIAP2	0.46	92	P2RX7	0.21	134	VPS41	0.10
9	PLCB1	3.04	51	FGF20	0.46	93	PARK7	0.21	135	WARS1	0.10
10	IL1B	2.94	52	mir-155	0.46	94	PRKN	0.21	136	AGRN	0.05
11	IFNG	2.88	53	PDE6G	0.46	95	SST	0.21	137	ARTN	0.05
12	PPP2CA	2.06	54	NFE2L2	0.41	96	TH	0.21	138	CALCA	0.05
13	PTGS2	1.80	55	NLRP3	0.41	97	TYR	0.21	139	CBX4	0.05
14	HLA-DRB1	1.60	56	TF	0.41	98	CHRNA4	0.15	140	CHMP2B	0.05
15	PAK4	1.54	57	APP	0.36	99	CHRNB2	0.15	141	DYNLT1	0.05
16	ABL1	1.49	58	HMGB1	0.36	100	CTSL	0.15	142	ENAH	0.05
17	IGF1	1.39	59	KLK6	0.36	101	ENDOG	0.15	143	GAK	0.05
18	CFL1	1.34	60	MPO	0.36	102	MT-CO3	0.15	144	LAG3	0.05
19	HSP90AA1	1.34	61	NR1H3	0.36	103	RAB11A	0.15	145	LRRK2	0.05
20	MMP2	1.34	62	NEDD4	0.36	104	SIRT2	0.15	146	mir-7	0.05
21	FOXO3	1.24	63	SNCA	0.36	105	TGM2	0.15	147	NRGN	0.05
22	MYD88	1.24	64	ADORA2A	0.31	106	TTR	0.15	148	OPTN	0.05
23	ICAM1	1.13	65	CLU	0.31	107	VDAC1	0.15	149	P2RX4	0.05
24	HMOX1	1.08	66	ESR2	0.31	108	ALAD	0.10	150	PLK2	0.05
25	HDAC6	1.03	67	GCG	0.31	109	CALB1	0.10	151	RAB1A	0.05
26	MMP3	0.98	68	GSN	0.31	110	GPX5	0.10	152	RGS6	0.05
27	TLR2	0.88	69	MCL1	0.31	111	CRYAB	0.10	153	RNF19A	0.05
28	CASP1	0.77	70	VAMP2	0.31	112	CTSK	0.10	154	RPH3A	0.05
29	PLD1	0.77	71	ABCA1	0.26	113	DGKZ	0.10	155	SDC3	0.05
30	SFN	0.77	72	CTSB	0.26	114	DNMT1	0.10	156	SLC11A1	0.05
31	NOX1	0.72	73	CTSD	0.26	115	FKBP1A	0.10	157	SLC6A3	0.05
32	ADRB2	0.62	74	DNM1	0.26	116	GPX5	0.10	158	SNCAIP	0.05
33	CAPN1	0.62	75	DYRK1A	0.26	117	H2AZ1	0.10	159	SNCB	0.05
34	CSNK2B	0.62	76	NR1H2	0.26	118	HTT	0.10	160	SNCG	0.05
35	FGF1	0.62	77	SNAP25	0.26	119	LAMP2	0.10	161	ST13	0.05
36	PSEN1	0.62	78	SOD1	0.26	120	LSM14A	0.10	162	SYN1	0.05
37	S1PR1	0.62	79	SQSTM1	0.26	121	OGA	0.10	163	SYN3	0.05
38	YWHAZ	0.62	80	STUB1	0.26	122	PINK1	0.10	164	TFEB	0.05
39	CXCL10	0.57	81	SUMO1	0.26	123	PPID	0.10	165	TXNIP	0.05
40	ESR1	0.57	82	YKT6	0.26	124	PSAP	0.10	166	USP8	0.05
41	HSPA4	0.57	83	CEBPD	0.21	125	RAB3A	0.10	167	USP13	0.05
42	MAPT	0.57	84	DNAJC13	0.21	126	SEPTIN4	0.10	168	UTRN	0.05

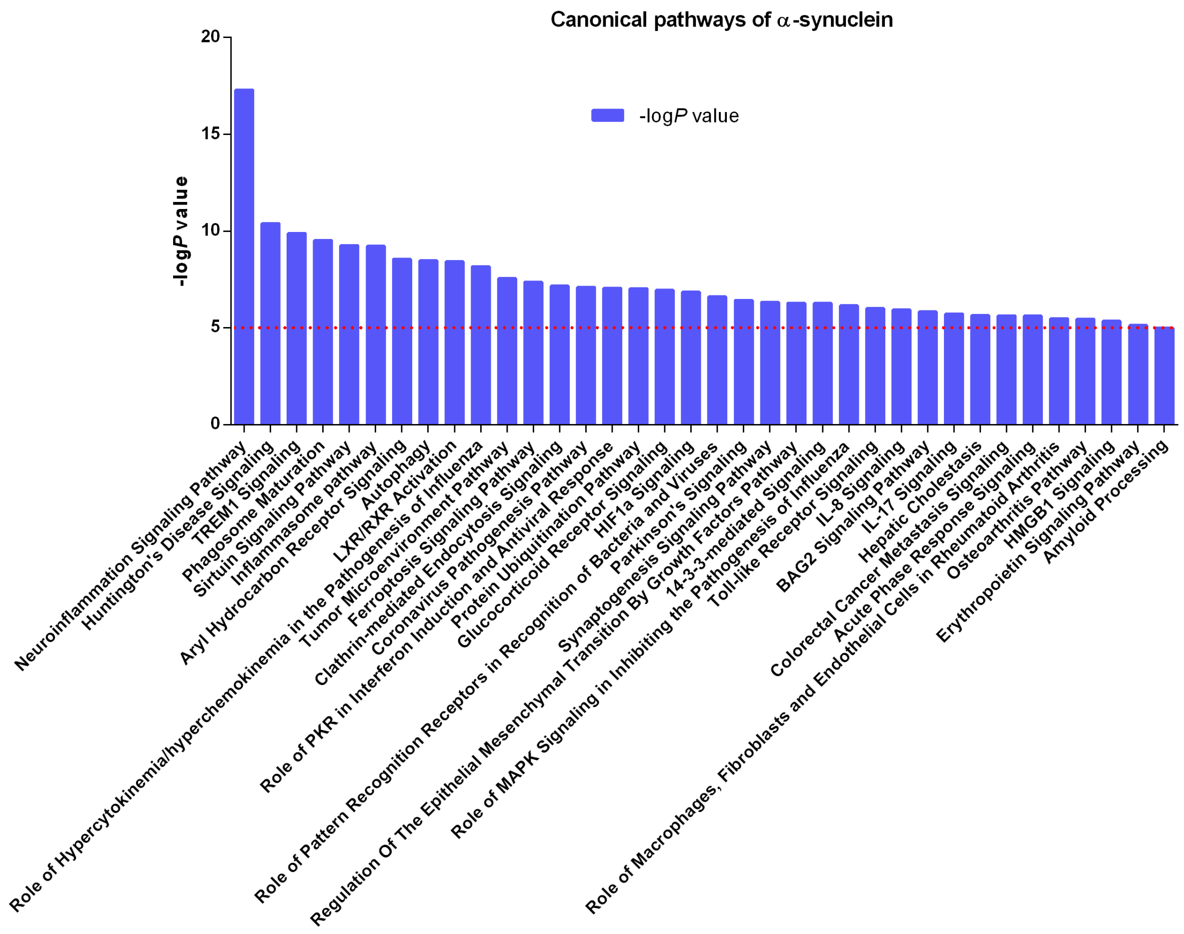

Figure S1. The major canonical signaling pathways of α-synuclein. The pathways with –log$P$ value >5.0 are only presented due to space constraints.

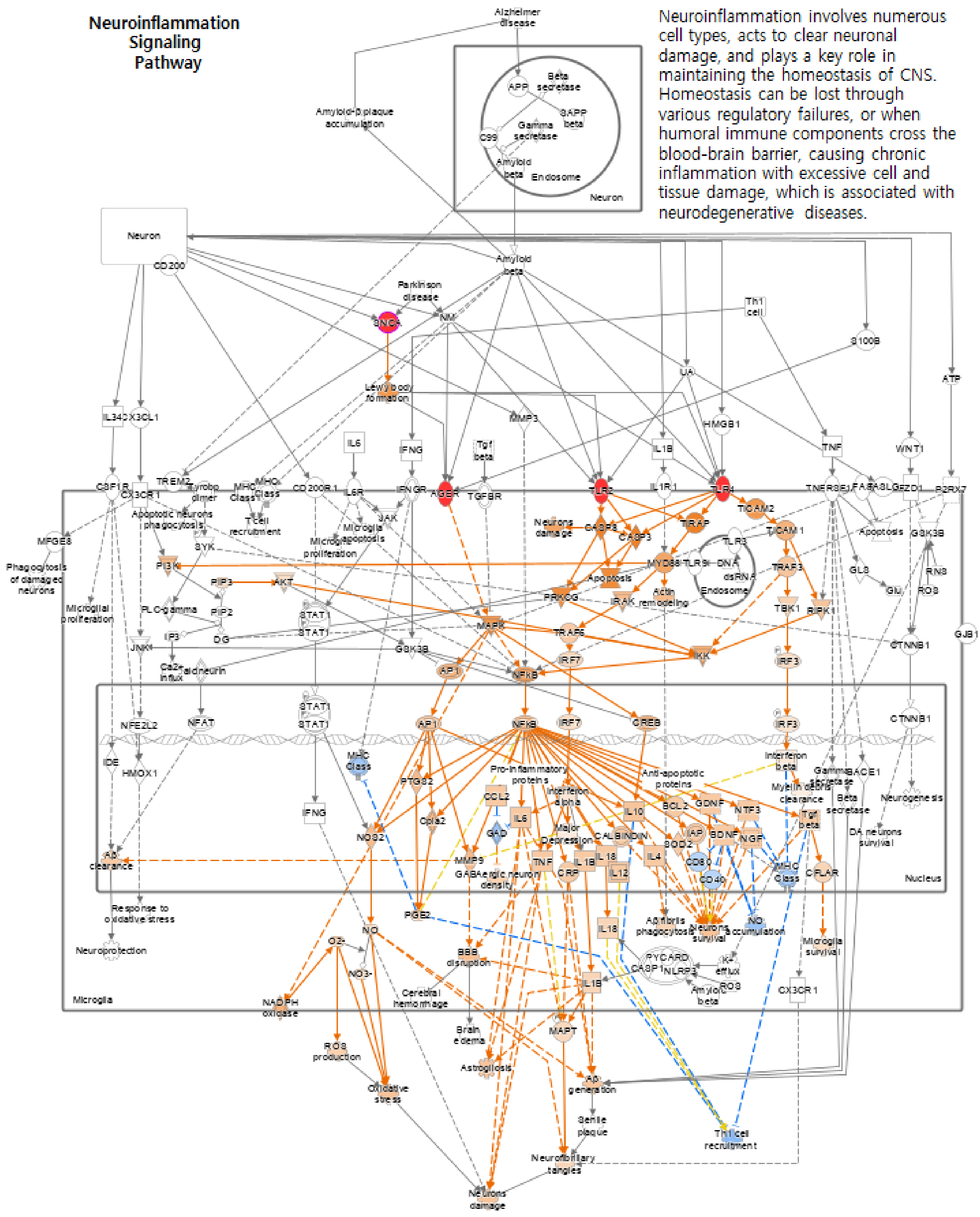

Figure S2. The role of α-synuclein in the neuroinflammation signaling pathway. Increased level of α-synuclein (SNCA) in Parkinson's disease leads to the formation of toxic Lewy body species, which causes death of dopaminergic neurons and neuroinflammation mediated by AGER, TLR2, and 4. AGER signal is facilitated by downstream molecule MAPK, which is further transduced by AP1, NFκB, and CREB. TLR2 signal is mediated by downstream molecules CASP-3, -8, and TIRAP while the TLR4 signal is mediated by downstream molecules CASP-3, -8, TIRAP, and TICAM2, which further activates MAPK, TRAF6, and IKK signaling cascades, leading to an increased level of canonical molecules AP1, NFκB, CREB, IRF7, and IRF3. Though both No effect of the increased activity of either APP or Aβ was predicted on the activity of α-synuclein (SNCA). Red color represents a higher degree of up-regulation or increase in the activity. Orange color indicates predicted activation, whereas blue color indicates predicted inhibition. Solid lines represent direct interaction, whereas dotted lines represent indirect interaction. Refer Fig. S3 prediction legend for more color details.

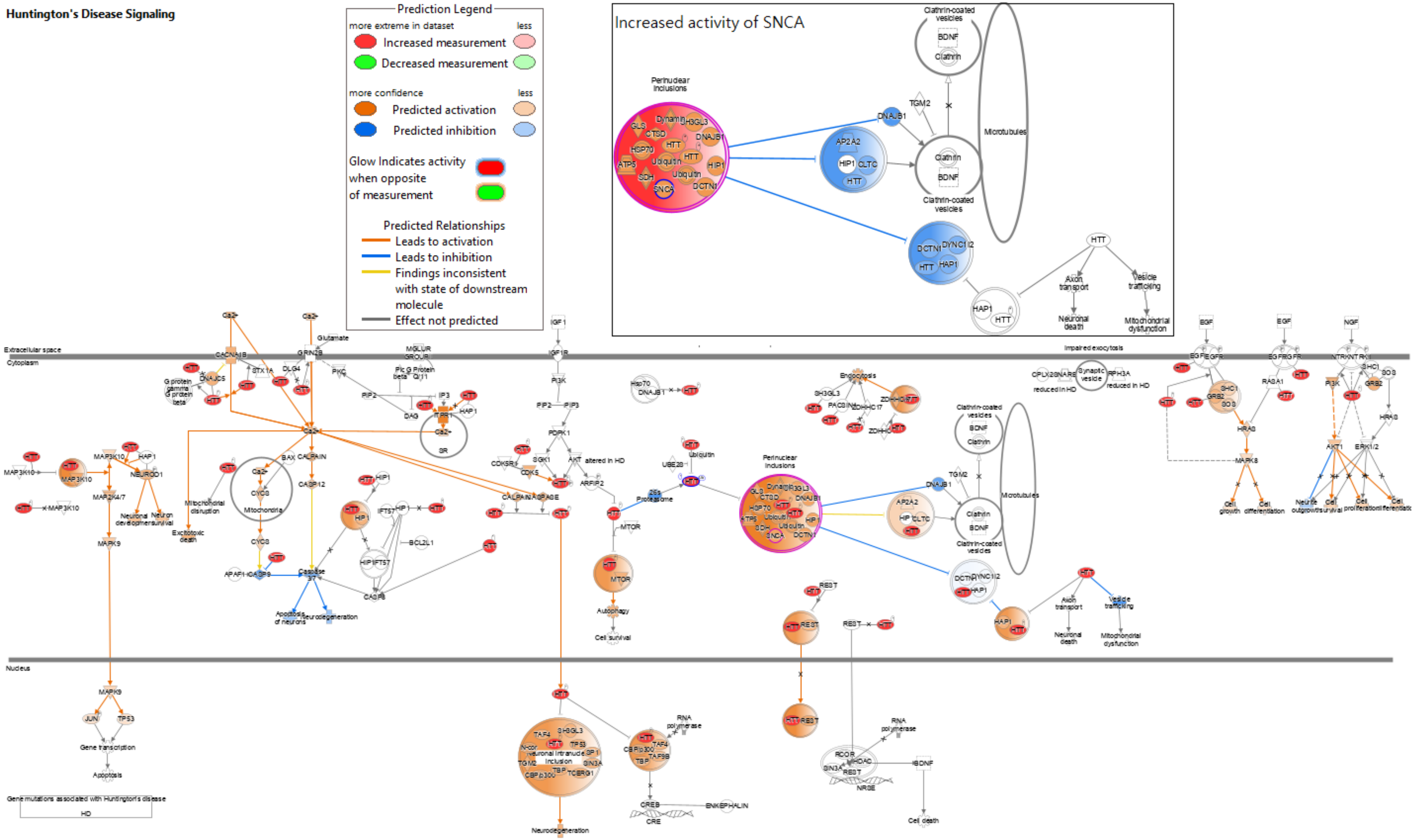

Figure S3. The role of α-synuclein in Huntington's disease. Increased level of HTT upregulates α-synuclein (SNCA) in the perinuclear inclusions of cytoplasm. Activation of α-synuclein (SNCA) suppresses the activity of DNAJB1 and DCTN1-DYNC1I2-HAP1-HD and AP2A2-CLTC-HD complexes, resulting in protein aggregation and impaired vesicular transport, respectively. The intensity of colors indicates the extent of up-or-down-regulation/activation or inhibition. Solid lines represent direct interaction, whereas dotted lines represent indirect interaction.

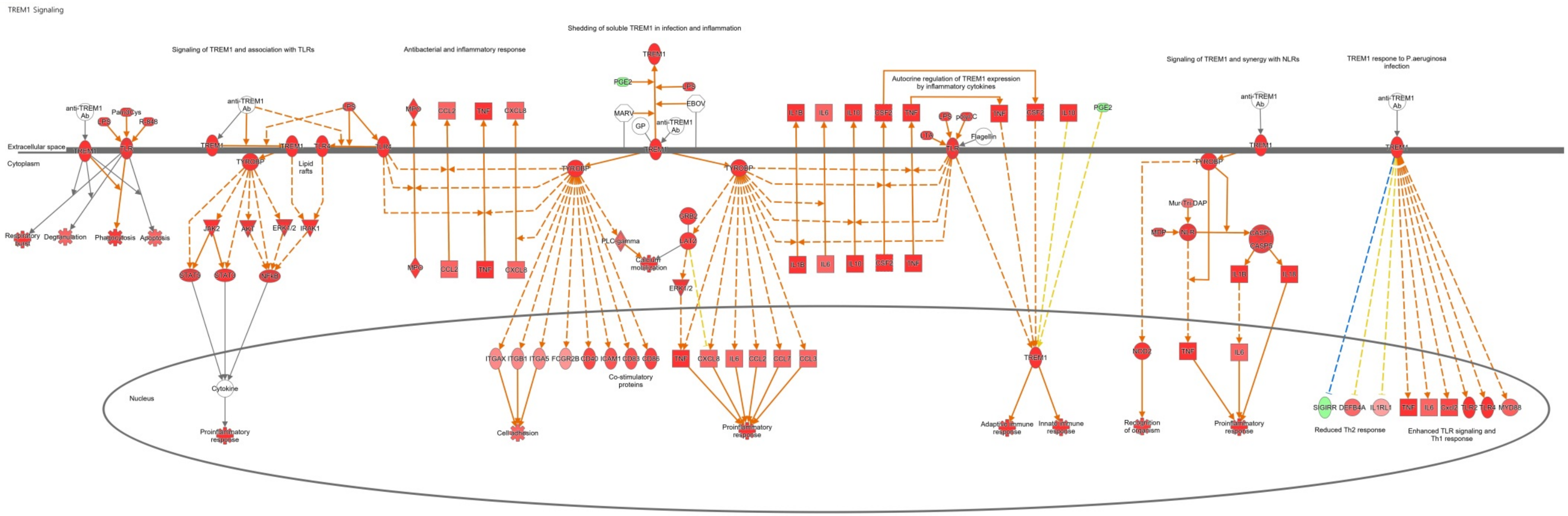

Figure S4. TREM1 signaling pathway of α-synuclein.

Red color represents a higher degree of up-regulation or increase in phosphorylation, whereas green color represents a higher degree of down-regulation or decrease in phosphorylation. Orange color represents predicted activation, whereas blue color represents predicted inhibition. The intensity of colors indicates the extent of up-or-down-regulation/activation or inhibition. Solid lines represent direct interaction, whereas dotted lines represent indirect interaction. Orange lines represent increased activity of downstream molecule upon activation or inhibition of upstream molecule with findings consistent with the state of downstream molecule. Blue lines represent decreased activity of downstream molecule upon activation or inhibition of upstream molecule with findings consistent with the state of downstream molecule. Yellow lines represent increased or decreased activity of downstream molecule upon activation or inhibition of upstream molecule with findings inconsistent with the state of downstream molecule.

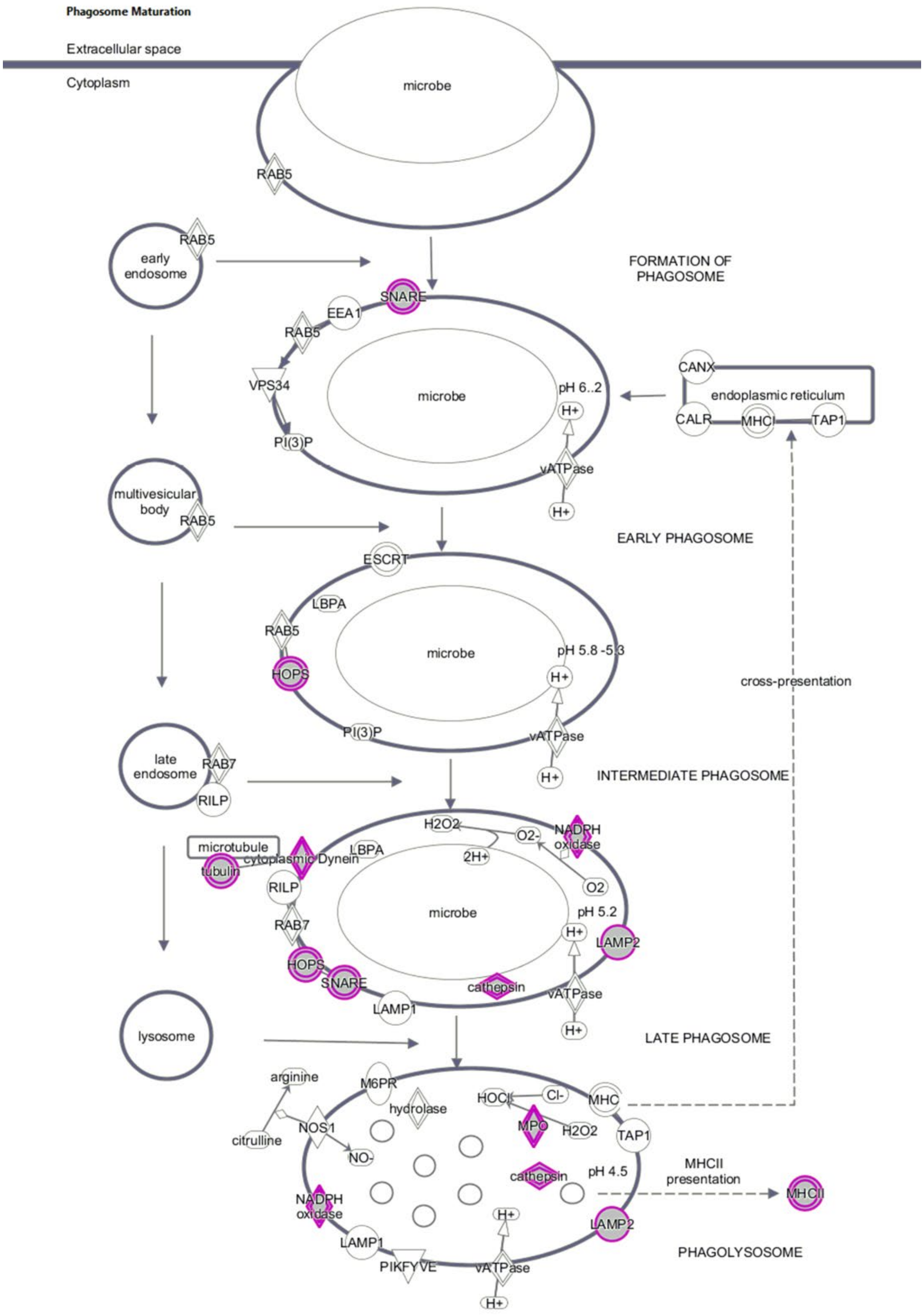

Figure S5. Phagosome maturation signaling pathway of α-synuclein.

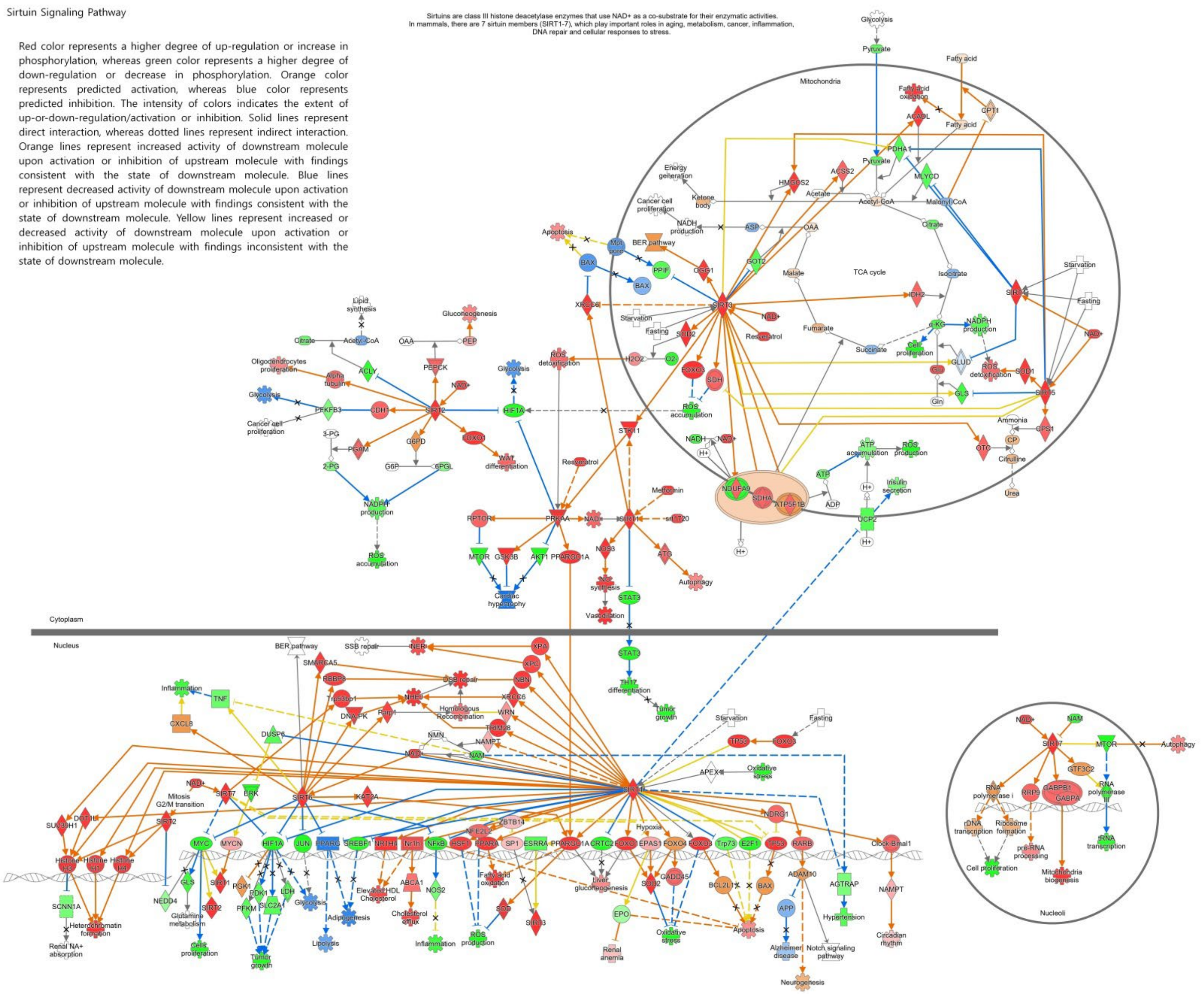

Figure S6. Sirtuin signaling pathway of α-synuclein.

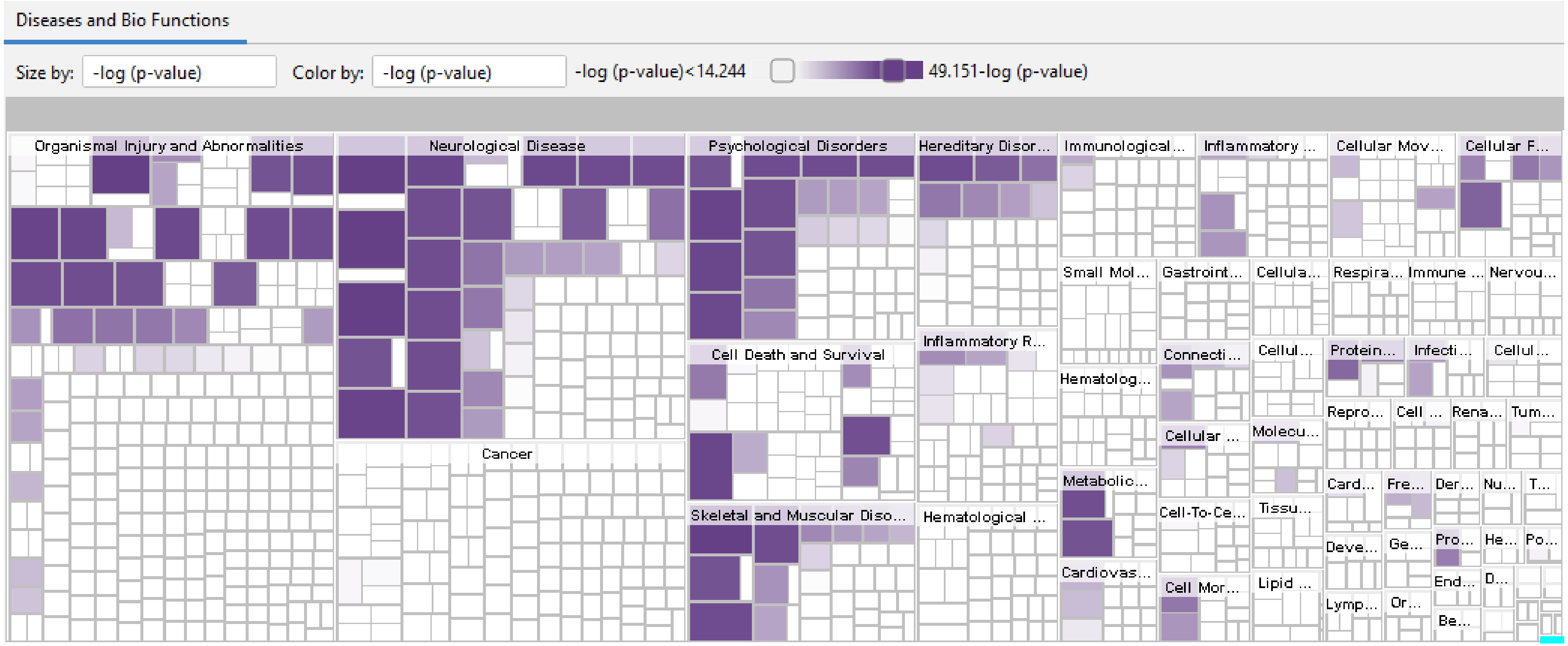

Figure S7. The heat map diagram exhibiting diseases and biological functions of α-synuclein.

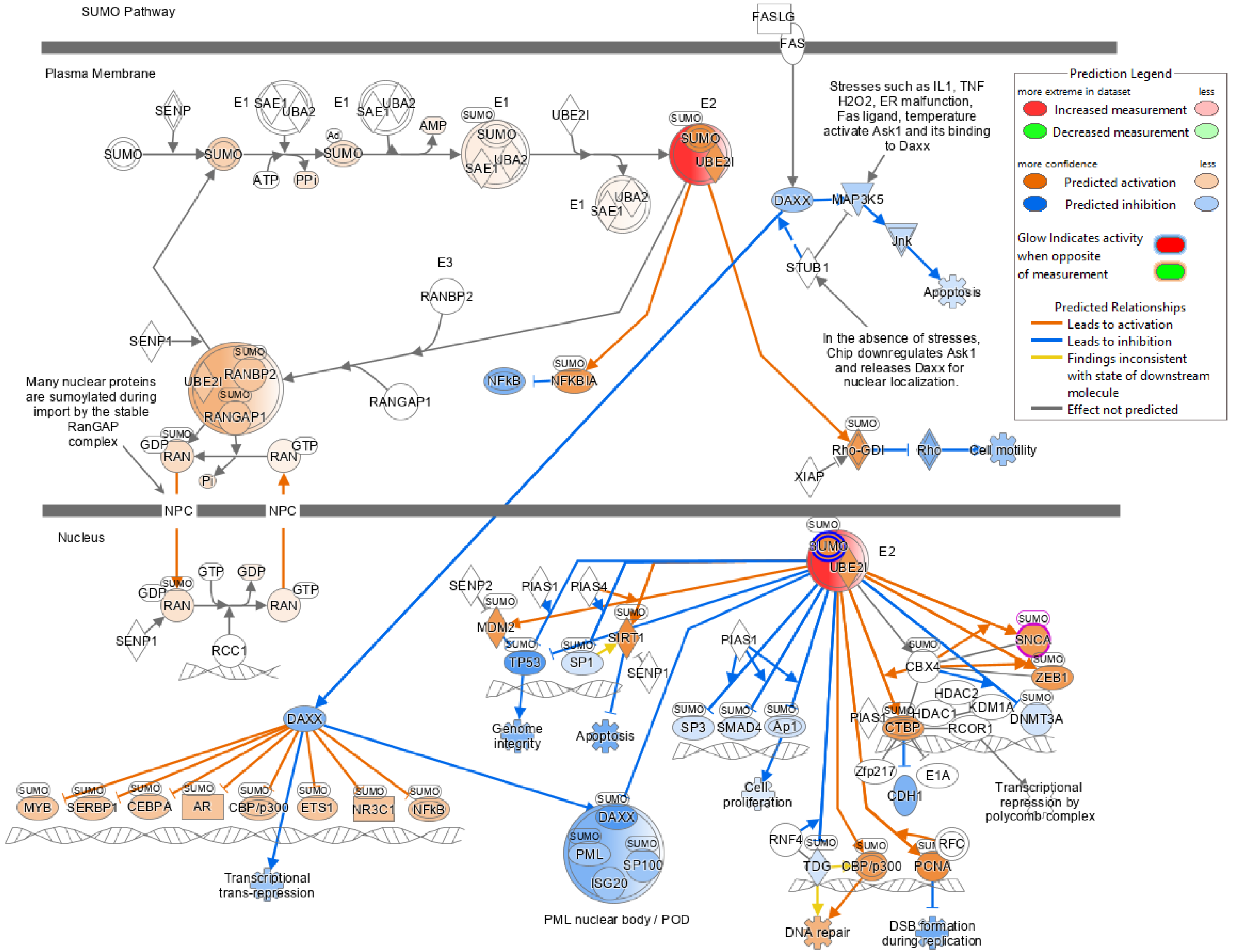

Figure S8. The role of α-synuclein in the sumoylation signaling pathway. Increased activity or activation of SUMO leads to activation of α-synuclein (SNCA), whereas inhibition of SUMO was associated with suppression of α-synuclein (SNCA) activity. The effect of activation or inhibition of α-synuclein (SNCA) activity was not predicted possibly due to the absence of any downstream α-synuclein (SNCA) effectors.

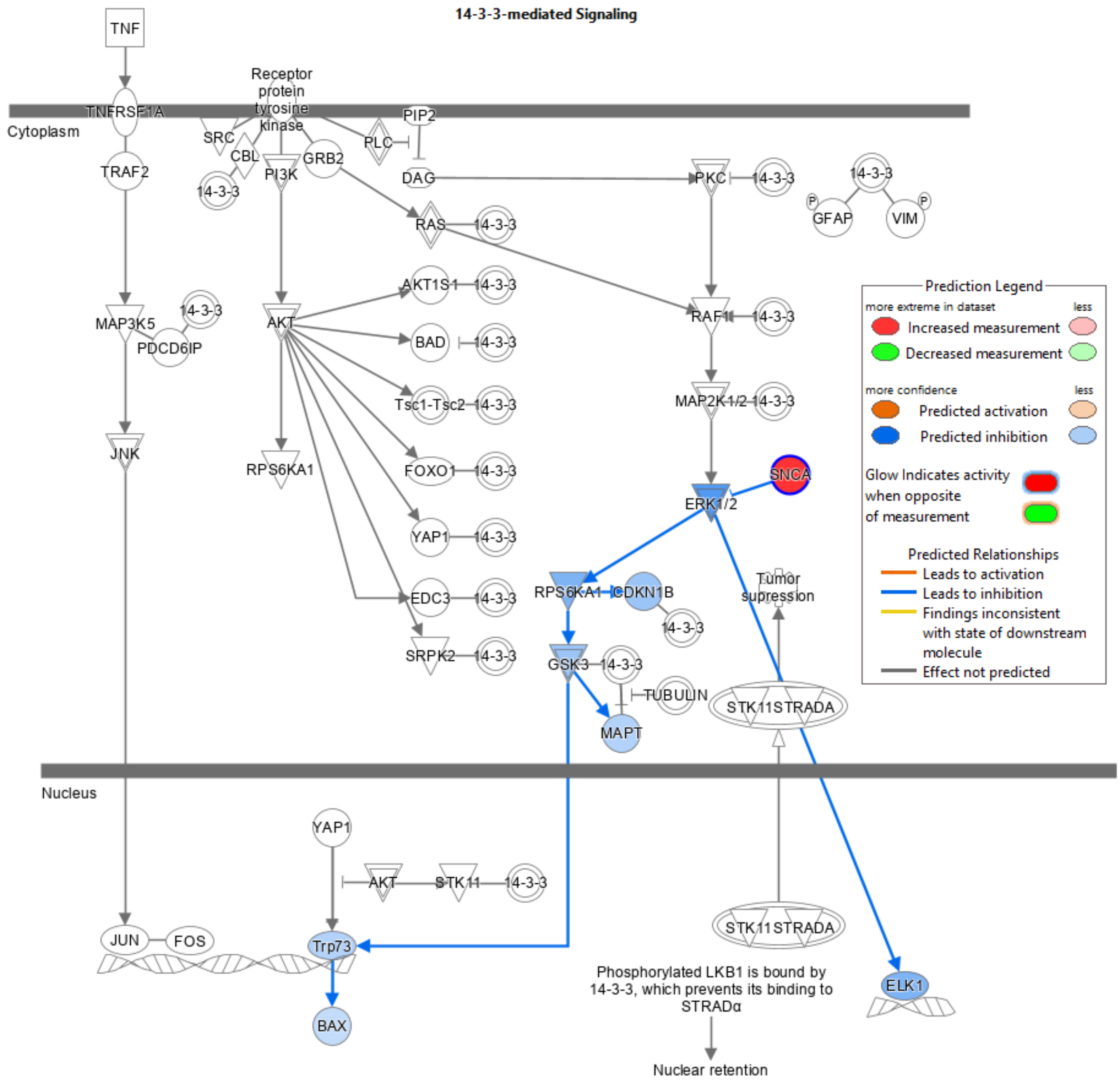

Figure S9. The role of α-synuclein in the 14-3-3-mediated signaling pathway. Increased activity of 14-3-3 leads to activation of downstream ERK1/2 signaling; however, no effect on α-synuclein (SNCA) activity was predicted, whereas increased activity of α-synuclein (SNCA) inhibits downstream ERK1/2 signaling.

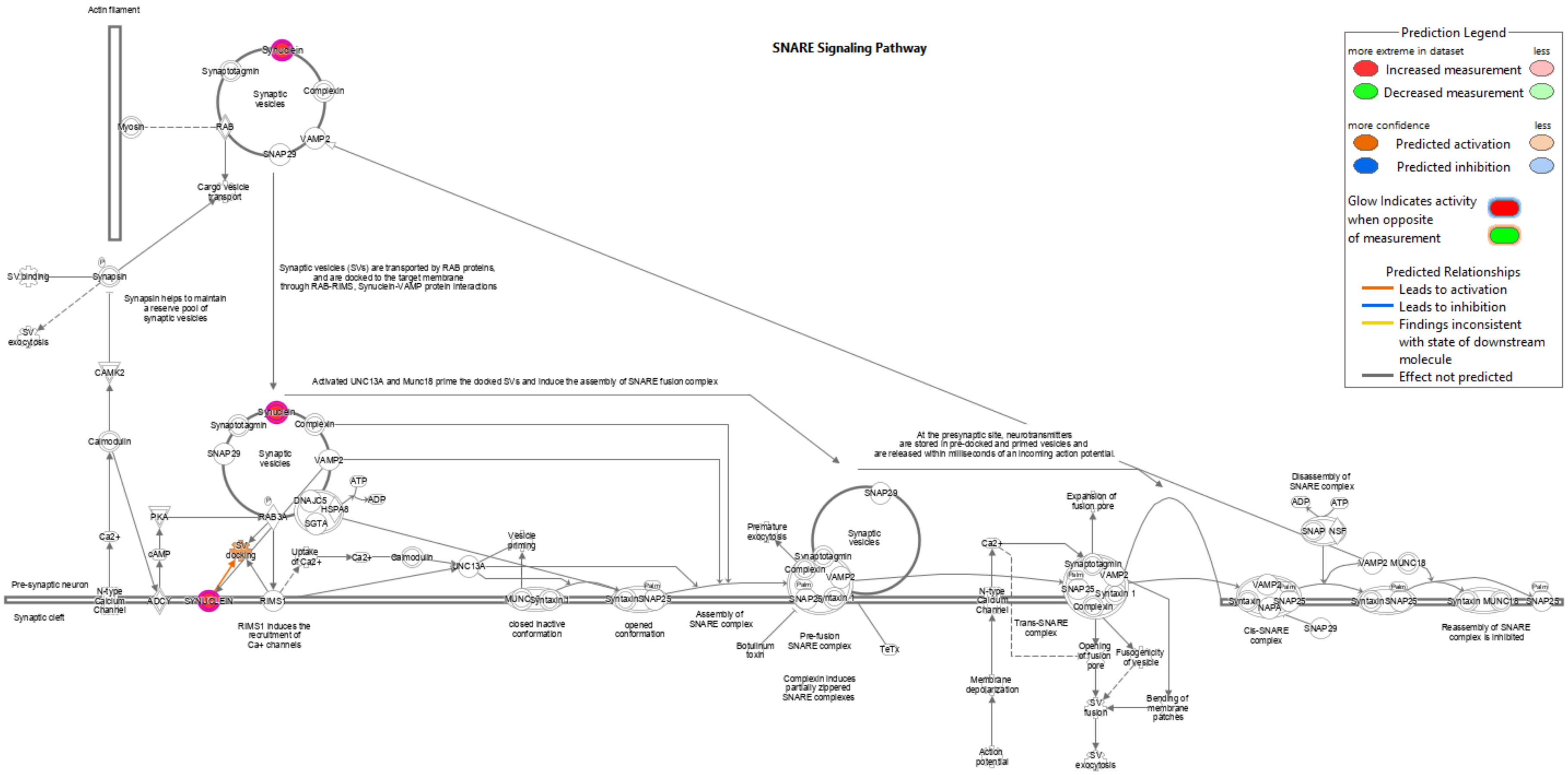

Figure S10. The role of α-synuclein in the SNARE signaling pathway. The triggering of α-synuclein in SNARE signaling enables docking of synaptic vesicle onto the presynaptic membrane that eventually leads to exocytosis of neurotransmitters. Furthermore, the activation of vesicle-associated membrane protein 2 (VAMP2 or synaptobrevin) also activates α-synuclein; however, the effect of individual activation of synaptotagmin, Ras-associated binding protein (RAB), synaptosomal-associated protein 29 (SNAP29), and complexin on the activity of α-synuclein was not predicted.

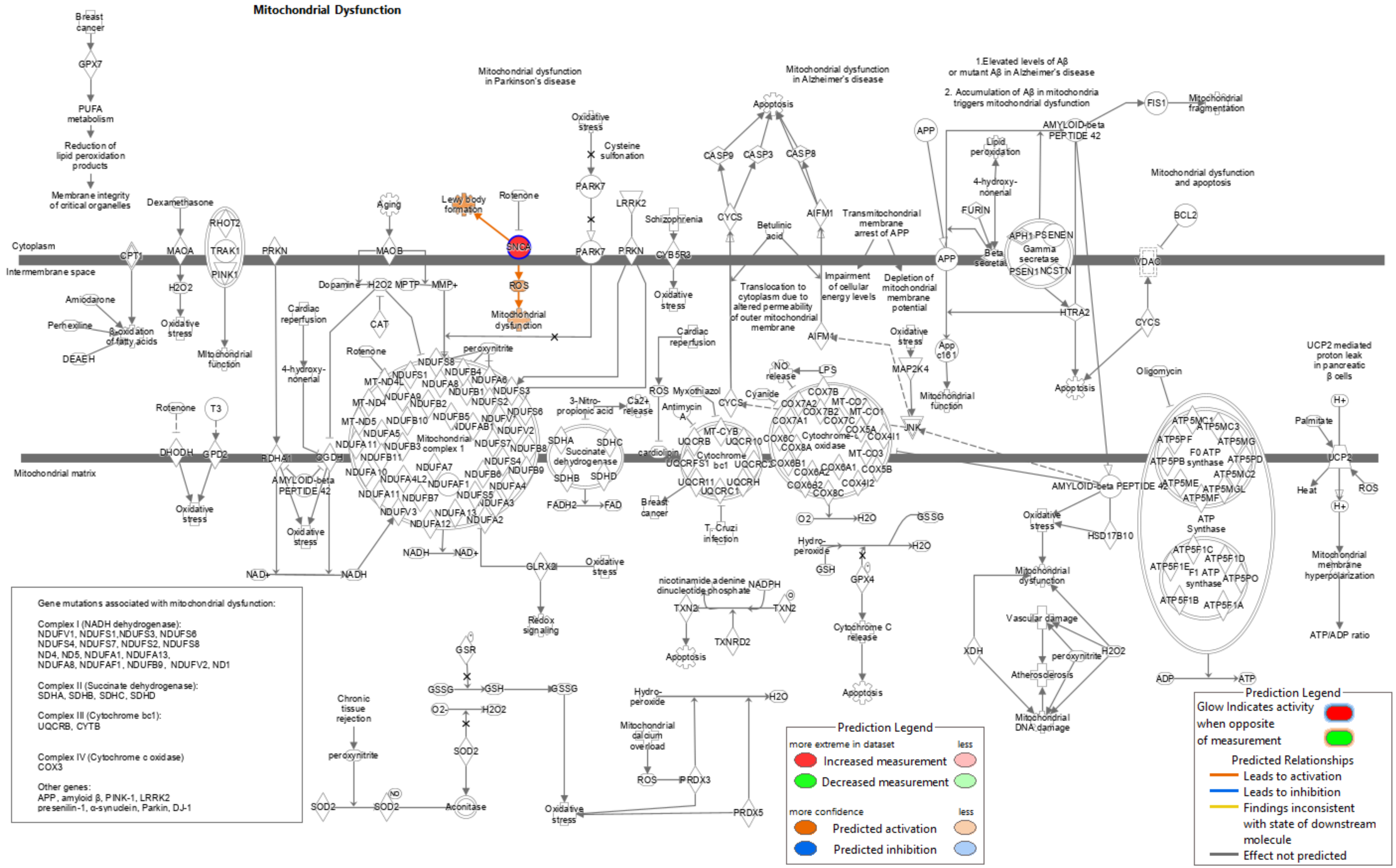

Figure S11. The role of α-synuclein in the mitochondrial dysfunction signaling pathway. Increased activity of α-synuclein (SNCA) leads to the formation of Lewy bodies and generates reactive oxygen (ROS) species that produce oxidative stress, resulting in mitochondrial dysfunction.

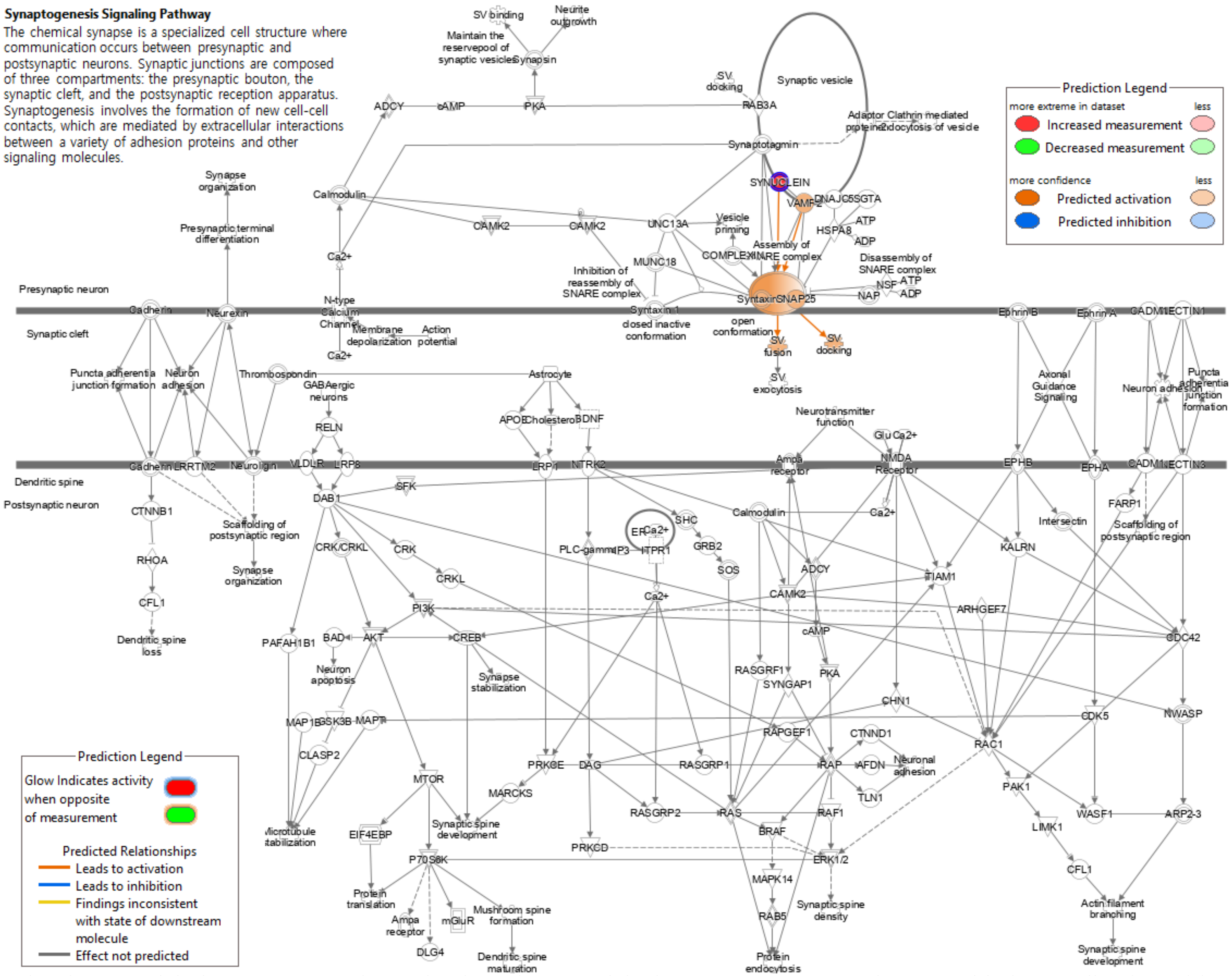

Figure S12. The role of α-synuclein in synaptogenesis. Activation of α-synuclein forms a SNARE complex assembly in coordination with vesicle-associated membrane protein 2 (VAMP2), and presynaptic membrane proteins syntaxin1, and SNAP25, which leads to synaptic vesicle docking, fusion, and exocytosis.
